# The causal relationship between 25-hydroxyvitamin D and serum lipids levels: A bidirectional two-sample mendelian randomization study

**DOI:** 10.1371/journal.pone.0287125

**Published:** 2024-02-14

**Authors:** Tianxiu Yin, Xiaoyue Zhu, Zhiliang He, Hexiang Bai, Chenye Shen, Ruoyu Li, Bei Wang

**Affiliations:** Department of Epidemiology and Health Statistics, School of Public Health, Southeast University, Nanjing, China; Università degli Studi di Milano, ITALY

## Abstract

Serum vitamin D levels were linked to lipid metabolism in observational studies, but the exact mechanism was unclear. Several studies have attempted to decipher the relationship between 25(OH)D and lipid levels. Conventional observational studies are vulnerable to confounding. Mendelian randomization (MR) analysis can better control for confounding factors and reverse causality, allowing for the inference of causal association. We, therefore, sought to use MR to investigate the possible causal relationship between 25(OH)D and blood lipid levels (HDL cholesterol, LDL cholesterol, triglycerides, and total cholesterol). A bidirectional two-sample Mendelian randomization (MR) was performed on data primarily from European ancestors. In addition, the potential causal effect of lipids on 25(OH)D was assessed by regressor-based multivariate magnetic resonance (MVMR). The single-nucleotide polymorphisms (SNPs) related to 25(OH)D were selected from a large-scale genome-wide association study (GWAS) database named IEU GWAS, and the SNPs associated with the four blood lipids were chosen from UK Biobank (UKB) lipid GWAS. When blood lipids were the outcome, the results of bidirectional two-sample MR demonstrated that 25(OH)D exhibited a negative causal association with TG, TC, and LDL-C: β = - 0.23, 95% CI = -0.28 to -0.19, *P*<0.001; β = - 0.16, 95% CI: - 0.30 to—0.03, *P* < 0.05; β = - 0.11, 95% CI: - 0.23 to 0, *P* < 0.05. There was no causal relationship between 25(OH)D and HDL-C (β = 0.05, 95% CI: - 0.11 to 0.20, *P* = 0.56). When setting blood lipids as exposure, TG and 25(OH)D, β = -0.13, 95% CI: - 0.15 to -0.10, *P* < 0.05; TC and 25(OH)D, β = -0.11, 95% CI: - 0.15 to -0.07, *P* < 0.05; HDL-C and 25(OH)D, β = 0.02, 95% CI: 0 to 0.03, *P* = 0.07; LDL-C and 25(OH)D, β = -0.08, 95% CI: - 0.11 to -0.05, *P* < 0.05). Our MVMR study also showed a significant relationship between genetically determined lipid traits and 25(OH)D levels (TG and 25(OH)D, *P* < 0.05; TC and 25(OH)D, *P* < 0.05). In all MR analyses, there was no horizontal pleiotropy (all *P* > 0.05), or statistical heterogeneity. The "Leave-one-out" sensitivity analysis confirmed the stability of our results. MR Studies have shown a bidirectional causal relationship between genetically-determined 25(OH)D levels and serum TG and TC levels. The findings have potential implications for etiological understanding and disease prevention.

## 1 Introduction

Vitamin D deficiency is common across the globe [1]. Inadequate vitamin D levels may contribute to a range of chronic conditions, including cardiovascular disease, stroke, and diabetes, in addition to being harmful for bone health [2–6]. Dyslipidemia, which refers to elevated levels of triglycerides (TG), total cholesterol (TC), and LDL cholesterol (LDL-C) and decreased levels of HDL cholesterol (HDL-C), has been identified as a significant risk factor for atherosclerosis and cardiovascular disease (CVD) [7]. A sensitive biomarker of vitamin D levels in the body is serum 25(OH)D [8]. Previous studies have shown a relationship between serum 25 (OH) D levels and blood lipids. But the results were inconsistent. The study results of Joshua Lupton et al. showed that serum 25 (OH) D was positively correlated with HDL-C and negatively correlated with LDL-C and TG in American adults [9]. MR Analysis by Xiao-Mei Mai et al. showed a positive causal relationship between serum 25 (OH) D and HDL-C, independent of total cholesterol, non-HDL-C, or triglycerides [10]. Yang Kaili et al. showed that serum 25 (OH) D in Chinese adults was negatively correlated with HDL-C, but not with TG, TC, and LDL-C levels [11]. Bea JW et al. showed that serum 25(OH)D level was negatively correlated with TG, but not with HDL-C [12].

Notably, several studies have discovered a significant relationship between seasonal vitamin D levels and lipid profiles, and research has suggested that having a higher BMI can lead to vitamin D deficiency [13, 14]. Zhe Lu et al. hypothesized that when lipid metabolism is disrupted, people are more likely to have vitamin D metabolism disorders. They carried out a two-sample Mendelian randomized study and discovered that elevated serum triacylglycerol, LDL, and HDL levels were all causally associated with vitamin D deficiency, but the results were significantly pleiotropic [15].

Observational epidemiological studies are limited in their ability to infer causality between circulating vitamin D and lipids due to residual confounding, other biases, and reverse causality [16, 17]. Single nucleotide polymorphisms (SNPs) associated with specific exposures are used as instrumental variables in Mendelian randomization (MR) studies to identify causality between risk factors (exposures) and disease (outcomes) [18]. SNPs are not associated with potential confounders due to the random distribution of genetic variation during meiosis and the natural causal effect of genetic variation on phenotypes, which makes MR Analysis less susceptible to confounders and reverse causality bias [19, 20].

In order to ascertain the causal connection between circulating serum 25(OH)D levels and serum lipid levels, we conducted a bidirectional two-sample MR Analysis using pooled statistics from publically available genome-wide association study (GWAS) data.

## 2 Materials and methods

### 2.1 Study design

**Fig 1:**
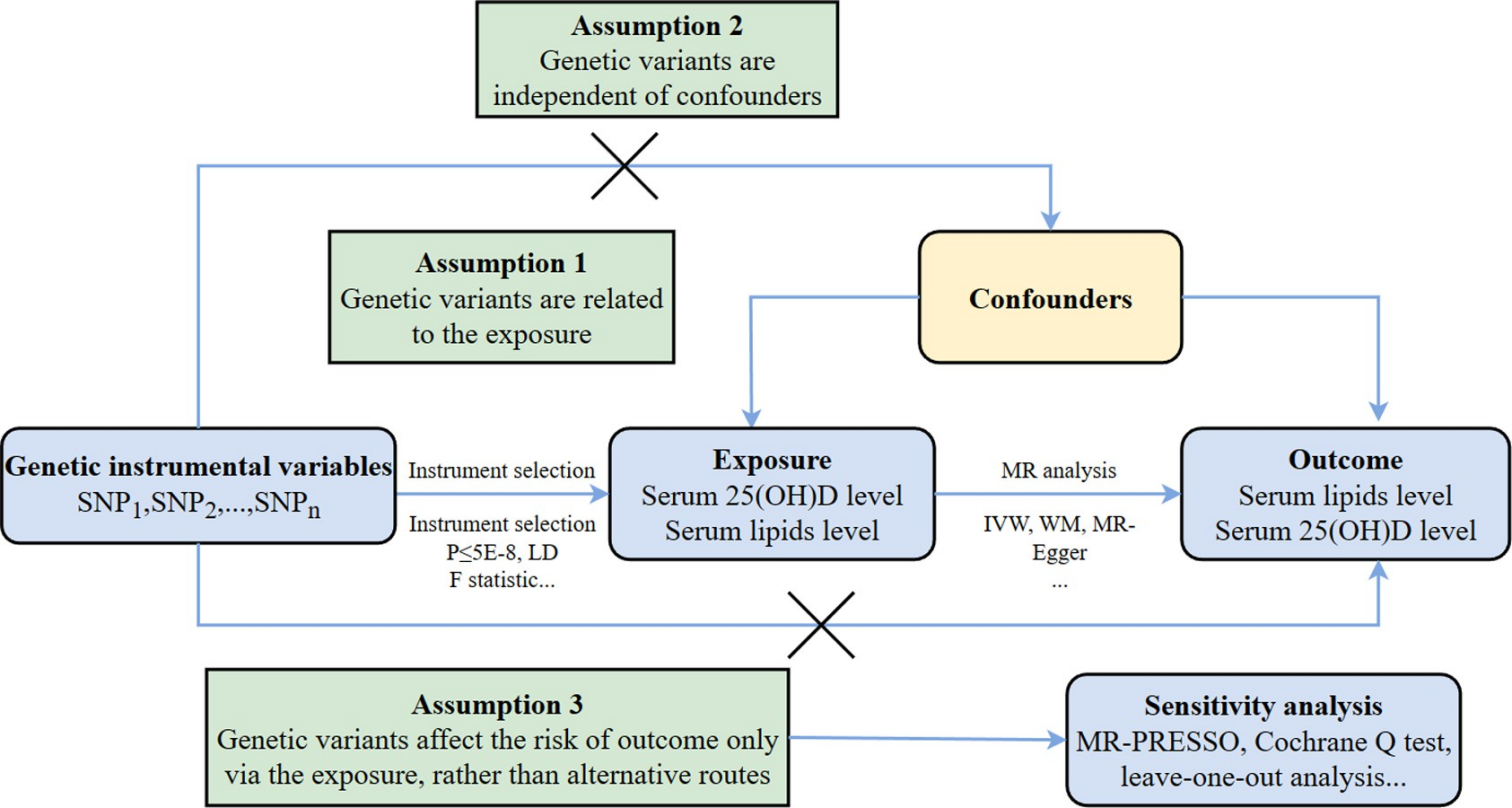
Schematics for the MR design. Abbreviation: MR, Mendelian randomization; SNP, single nucleotide polymorphism; LD, linkage disequilibrium; IVW, Inverse Variance Weighted; WM, Weighted Median; PRESSO, Pleiotropy REsidual Sum and Outlier.

We report MR Studies following the reporting guidelines for enhanced observational epidemiological studies using Mendelian randomization (STROBE-MR) [21]. The framework of the two-sample MR Study is shown in Fig 1. MR Analysis was performed to estimate the causal relationship between serum 25(OH)D and lipid levels. We chose SNPs as the genetic instrument and conducted the MR analysis using the three fundamental MR design assumptions: 1) Relevance, i.e., genetic IVs are strongly associated with the exposure (Assumptions Ⅰ); 2) Independence, i.e., IVs are independent of any confounders (Assumptions Ⅱ); and 3) Exclusiveness, i.e., IVs do not affect the outcome directly, only possibly indirectly via the exposure (Assumptions Ⅲ) [19]. Robust MR methods with different model assumptions were utilized, such as inverse variance weighted (IVW), MR-Egger, weighted mode, Weighted median. Subsequently, we performed a series of analyses to identify potential pleiotropy and heterogeneity. We investigated reverse causation further by performing an MR study using blood lipids as the exposure and 25(OH)D as the outcome. To account for the documented horizontal pleiotropy between lipids, we conducted a multivariable MR (MVMR). Since all analyses in our study were based on publicly available GWAS datasets, ethics approval was not required.

### 2.2 Genome-Wide association study datasets

The IEU GWAS database, a freely accessible resource (https://gwas.mrcieu.ac.uk/datasets/), provided GWAS for 25(OH)D and four lipids. Related to serum 25 (OH) D level of SNPs from GWAS meta-analysis (https://gwas.mrcieu.ac.uk/datasets/ieu-b-4808/), including 441,291 subjects of European descent. For GWAS data of TG, HDL-C, and LDL-C, lipid GWAS from UKB (ID: ieu-b-111 based on TG of 441,016 subjects of European ancestry, ieu-b-109 based on HDL-C of 403,943 subjects of European ancestry, and ieu-b-110 based on LDL-C of 440,546 subjects of European ancestry), aggregated statistics for TC were obtained from the GWAS meta-analysis (https://gwas.mrcieu.ac.uk/datasets/met-d-Total_C /), including 115,078 subjects of European descent.

### 2.3 Generation of genetic instruments

SNPs associated with 25(OH)D were selected as IVs (*P* < 5 × 10−^8^). We clumped the instrumental variables (r ^2^ < 0.01, windows size = 5000 kb) to remove the SNPs with strong linkage disequilibrium (LD), since these may cause biased results. For meaningful SNPs, the minor allele frequency (MAF) threshold was set at 0.01. Palindromic SNPs are removed from instrumental variables. In addition, we chose SNPs related to four blood lipids and used the same strategy to see if HDL-C, LDL-C, TG, and TC influenced serum 25(OH)D levels. We calculated the F statistic to assess whether there was a weak instrumental bias. If the F statistic was more than 10, the link was thought to be strong enough to avoid weak instrument bias [22].

### 2.4 Statistical analysis

This study applied a variety of complementary methods, including inverse variance weighting (IVW) method, MR-Egger regression, weighted median (WM) method and weighted model regression, to study the causal relationship between exposure and outcome.

Specifically, fixed effects or random effects IVW methods serve as the primary analysis for causal estimation, which will provide the most accurate results when all IVs are valid [23]. Median weights can be estimated when 50% IV is not valid [24]. Although the MR-Egger method has low statistical power, it pro-vides estimates after adjusting for multiple effects [25]. These two methods are relatively robust to horizontal pleiotropy at the sacrifice of statistical power. Furthermore, by grouping the SNPs into subgroups based on the similarity of causal effects, the weighted mode technique may analyze the causal linkage of the subset with the greatest number of SNPs [26].

In addition, MR Pleiotropy RESidual Sum and Outlier (MR-PRESSO) tests were applied to detect potential horizontal multiplicity and correct it by removing outliers [27]. The Cochrane’s Q test was used to assess heterogeneity among SNPs in the IVW approach. The random effects IVW test was employed where there was heterogeneity (*P*<0.05) to generate a more cautious but reliable estimate. Finally, a retention analysis was conducted to ensure the reliability of the affiliation between SNPs and exposure and to determine whether any SNPs were responsible for the significant results. To eliminate potential bias induced by numerous exposures on the outcome when selecting blood lipids as exposure, multivariable MR analysis (MVMR) based on IVW mothed was utilized in this research to identify independent SNPs and compute an adjusted estimate [28].

All analysis was performed using the "TwoSampleMR" and "MRPRESSO" packages in R (version 4.0.3).

### 2.5 Sensitivity analysis

Additionally, heterogeneity was assessed by Cochran’s Q test. MR–Egger regression was used to detect pleiotropy. Furthermore, to assess the reliability and stability of MR data, this study performed a sensitivity analysis utilizing the "leave-one-out" sensitivity test. All statistical analyses were performed with the “Two-Sample MR” package in R (version 4.0.3). The results were statistically significant when *P* < 0.05.

## 3 Results

### 3.1 IVs selection for mendelian randomization analysis

To investigate the causal relationship between 25(OH)D and TG, TC, HDL-C and LDL-C, we selected 134, 142, 146 and 141 genome-wide significant variants of 25(OH)D (*P* < 5×10−^8^) as independent IV (r ^2^ < 0.01). In reverse MR, 250, 53, 267, and 145 significantly associated SNPs were identified between TG, TC, HDL-C, LDL-C and 25(OH)D. For these IVs, *F* > 10 indicates that the possibility of weak instrumental variable bias is small. The supplementary data sheets show the basic information of the selected SNPS for MR Analysis.

### 3.2 The casual effect of 25(OH)D to four types of blood lipids

**Fig 2:**
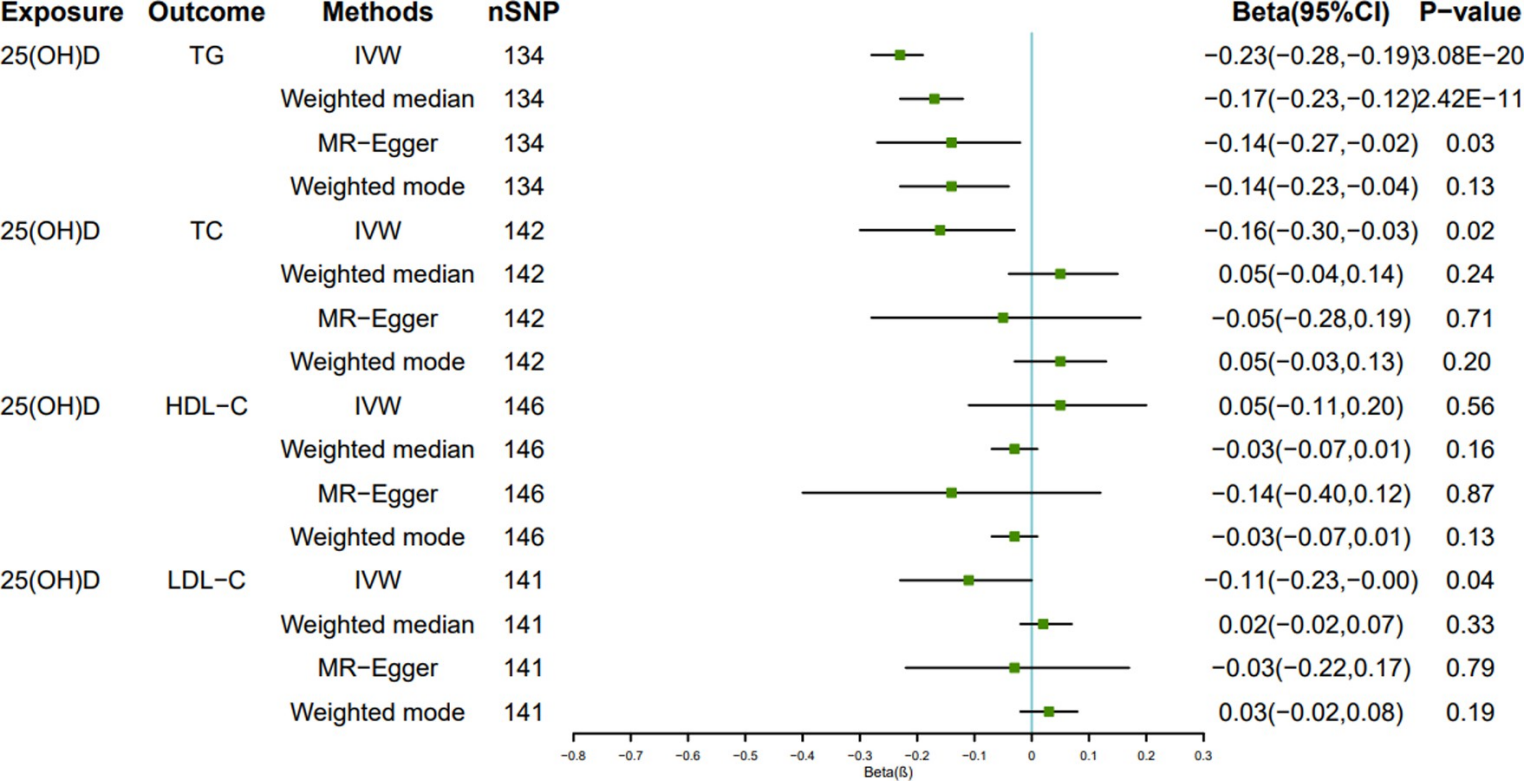
Forest plot of the causal effect of Mendel randomized (MR) analysis of serum 25(OH)D levels on lipid levels.

**Fig 3:**
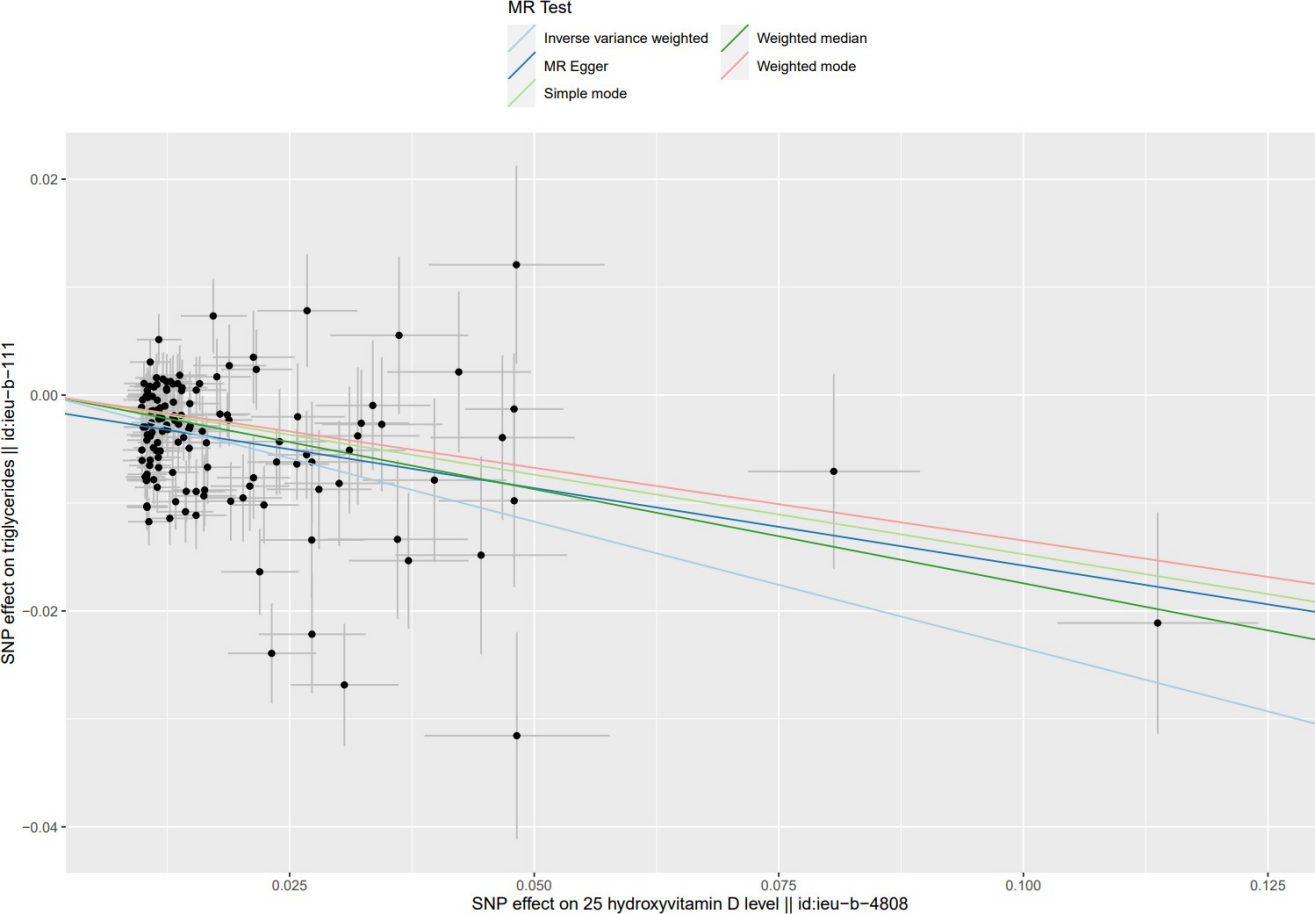
Scatter plot of causal effect of Mendel randomized (MR) analysis of serum 25(OH)D levels on TG levels.

**Fig 4:**
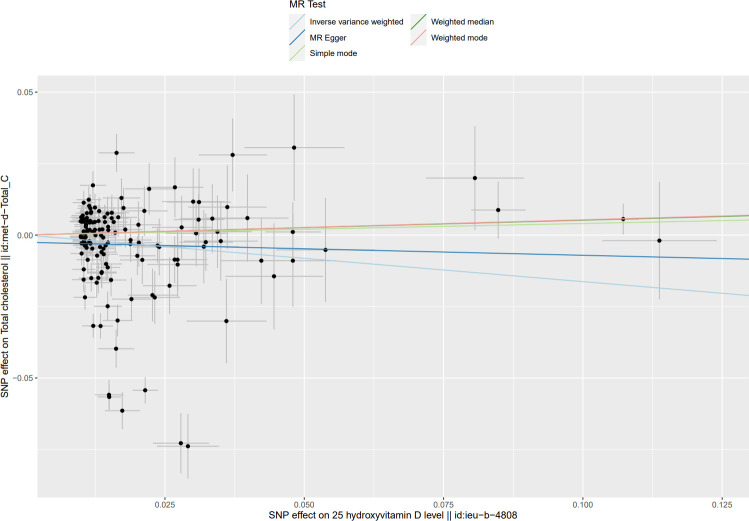
Scatter plot of causal effect of Mendel randomized (MR) analysis of serum 25(OH)D levels on TC levels.

**Fig 5:**
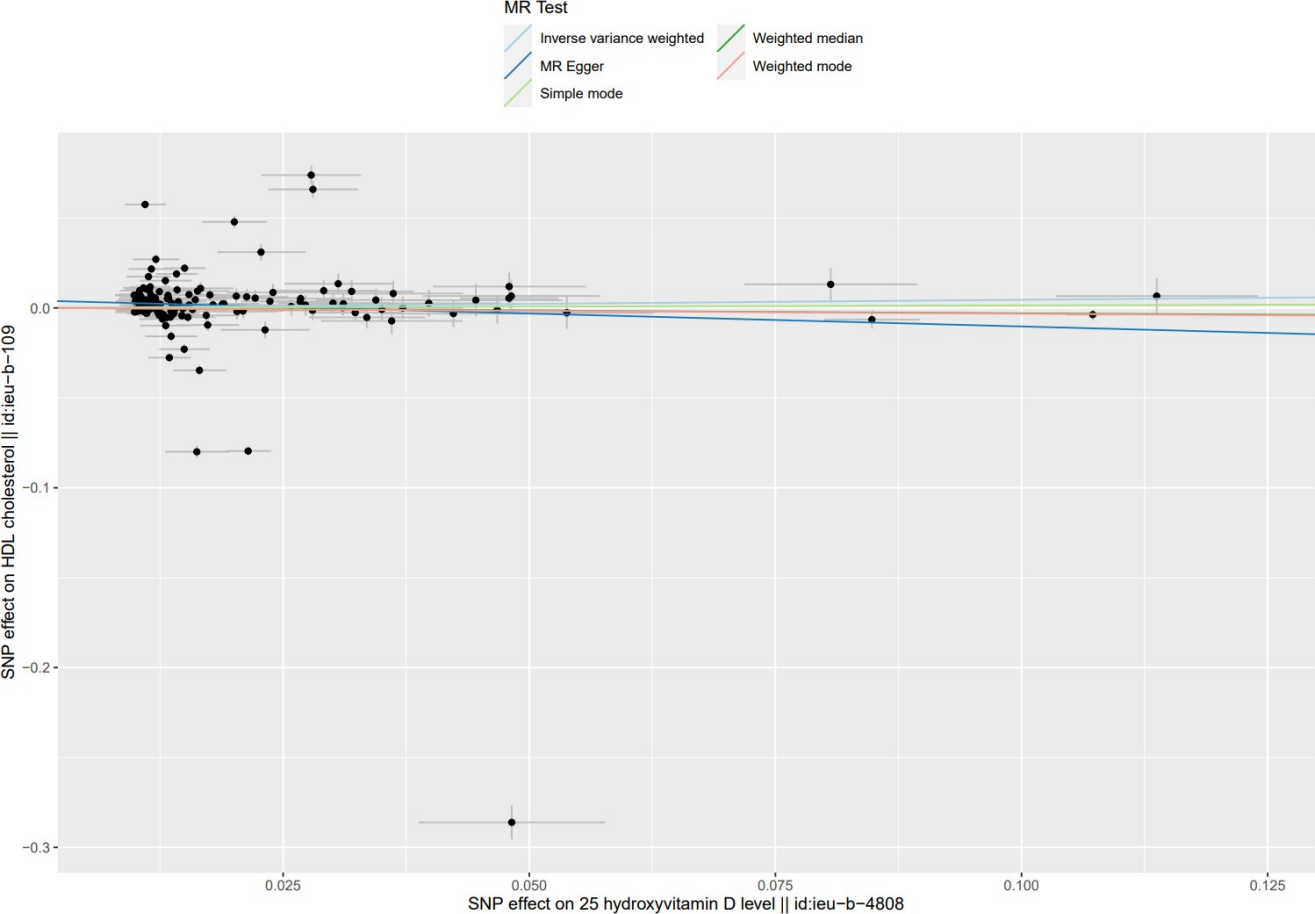
Scatter plot of causal effect of Mendel randomized (MR) analysis of serum 25(OH)D levels on HDL-C levels.

**Fig 6:**
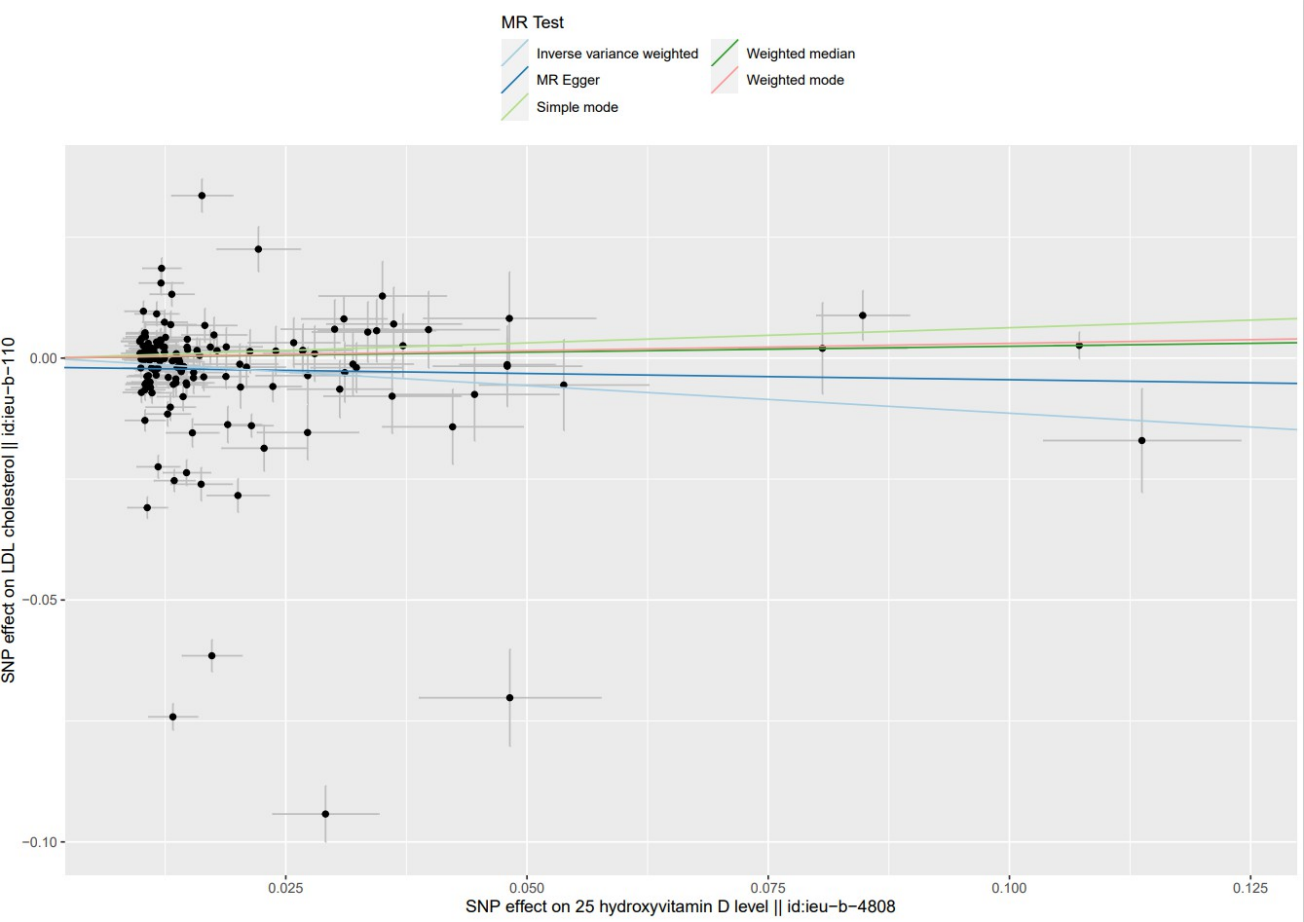
Scatter plot of causal effect of Mendel randomized (MR) analysis of serum 25(OH)D levels on LDL-C levels.

The MR Results of 25(OH)D on four kinds of lipids are shown in Fig 2 and reported as beta (β). The Cochrane’s Q test showed heterogeneity, but the MR-PRESSO test and MR-Egger regression did not detect any level of pleiotropy between the instrument SNPs, so the causal relationship between 25(OH)D and four kinds of blood lipids were determined using the random effects model of IVW method. Serum 25(OH)D level was negatively correlated with TG, TC, and LDL-C levels: β = - 0.23, 95% CI = -0.28 to -0.19, *P*<0.001; β = - 0.16, 95% CI: - 0.30 to—0.03, *P* < 0.05; β = - 0.11, 95% CI = - 0.23 to 0, *P* < 0.05. The results showed no causal relationship between serum 25(OH)D level and HDL-C level: β = 0.05, 95% CI: - 0.11 to 0.20, *P* = 0.56. Scatter plots of the causal relationship between 25(OH)D and lipid levels are shown in Figs 3–6.

A series of sensitivity tests including Cochrane’s Q test, leave one analysis, MR-Egger intercept and funnel plot were performed to assess the accuracy of the positive estimates. The *P*-values of all analyzed MR-Egger intercepts were all greater than 0.05, indicating that horizontal polymorphism did not exist in our study (Table 1). However, Cochrane’s Q test detected heterogeneity between 25(OH)D and TG(Q = 360.29, *P*<0.05), 25(OH)D and TC(Q = 1091.15, *P*<0.05), 25(OH)D and HDL-C (Q = 6073.34, *P*<0.05), 25(OH)D and LDL-C (Q = 2620.14, *P*<0.05). Although heterogeneity was detected in the results, it did not affect the robustness of the MR Estimates in this study because we used an IVW analysis under the multiplicative random effects model in the study, which would have balanced the heterogeneity. Additionally, the remaining analysis was unable to identify a single SNP with a biased effect on IVW (S1–S4 Figs). The funnel plot is symmetric, illustrating the reliability of the results (S5–S8 Figs).

**Table 1:** Heterogeneity and horizontal pleiotropy analyses between 25(OH)D and blood lipids.

Exposure	Outcome	Cochran Q test		MR-Egger Intercept test	
		Q value	*P*	MR-Egger Intercept	*P*
25(OH)D level	TG	360.29	<0.05	-0.001	0.131
25(OH)D level	TC	1091.15	<0.05	-0.003	0.237
25(OH)D level	HDL-C	6073.34	<0.05	0.004	0.082
25(OH)D level	LDL-C	2620.14	<0.05	-0.002	0.272

### 3.3 Reverse MR analysis

**Fig 7:**
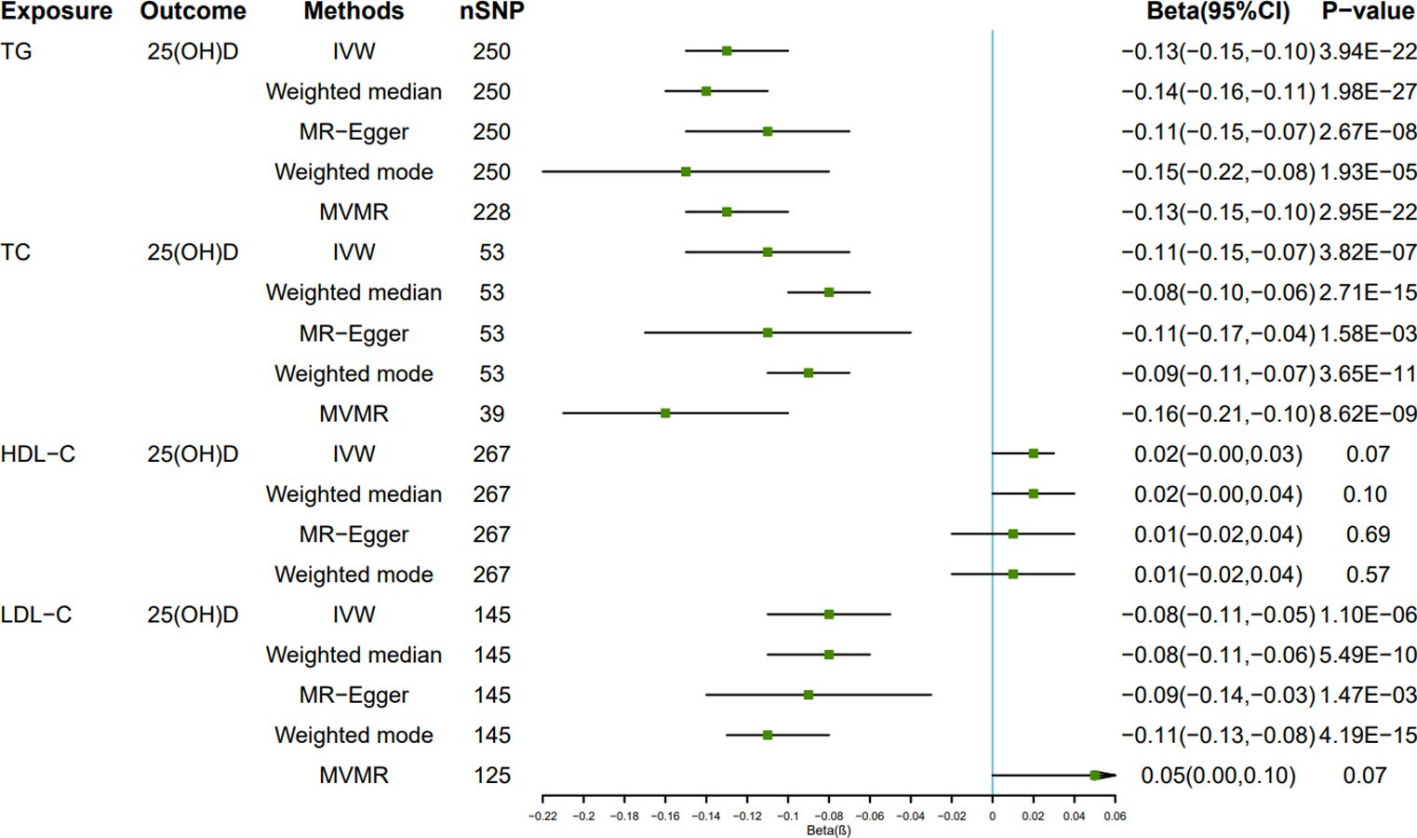
Forest plot of the causal effect of Mendel randomized (MR) analysis of lipid levels on serum 25(OH)D levels.

In reverse MR, levels of serum lipids were the exposure factor, and the level of 25(OH)D was the outcome variable. In univariable MR analysis, we simply tested the causation between each lipid and 25(OH)D. However, in MVMR analysis, we included the significant risk factors (TC, TG and LDL-C) from the univariable analysis. The univariable MR Results of four kinds of lipids on 25(OH)D are depicted in Fig 7 and reported as beta (β). Univariate MR results revealed that TG, TC, and LDL-C were all negatively causally correlated with 25(OH)D: IVW β = - 0.13, 95% CI = -0.15 to -0.10, *P*<0.001; IVW β = - 0.11, 95% CI: - 0.15 to—0.07, *P* < 0.05; IVW β = - 0.08, 95% CI = - 0.11 to -0.05, *P* < 0.05. The relationship between HDL-C and genetically predicted 25(OH)D was not statistically significant: IVW β = 0.02, 95% CI = 0 to 0.03, *P* = 0.07. Scatter plots are shown in S9–S12 Figs.

Cochran Q-value indicated that there was heterogeneity between the IVs extracted from HDL cholesterol, LDL cholesterol, TC and TG (Table 2), so we used a random effects model to estimate the MR effect size (Fig 7). Leave-one-out analysis, which can prevent horizontal pleiotropy, was conducted as a result of a single SNP based on the idea that removing one SNP at a time sequentially (S13–S16 Figs). Funnel plots are presented in S17–S20 Figs.

**Table 2:** Heterogeneity and horizontal pleiotropy analyses between 25(OH)D and blood lipids.

Exposure	Outcome	Cochran Q test		MR-Egger Intercept test	
		Q value	*P*	MR-Egger Intercept	*P*
TG	25(OH)D	1724.94	<0.05	-0.001	0.334
TC	25(OH)D	808.80	<0.05	-0.0001	0.962
HDL-C	25(OH)D	558.68	<0.05	0.0003	0.416
LDL-C	25(OH)D	1005.22	<0.05	0.0002	0.801

Given correlation among lipid-related characteristics, we conducted the multivariable MR analysis. The results of the MVMR analysis showed that TG (β = - 0.13, 95% CI: - 0.15 to—0.10, *P* < 0.05) and TC (β = - 0.16, 95% CI: - 0.21 to—0.10, *P* < 0.05) had a significant negative causal effect on 25(OH)D, which was consistent with the findings of the univariate analysis. There was no correlation between LDL-C and 25(OH)D (β = 0.05, 95% CI: 0 to 0.10, *P* = 0.07).

## 4 Discussion

Vitamin D deficiency has been linked to a variety of diseases, including cardiovascular disease [29, 30]. Dyslipidemia, characterized by elevated TG, TC, LDL-C and decreased HDL-C, has been identified as a risk factor for CVD [31, 32]. Vitamin D may have an impact on cardiovascular health by affecting blood lipids. The risk of vitamin D deficiency may also be increased by dyslipidemia, according to other studies. In earlier studies, the academic community focused more on the connection between dyslipidemia and vitamin D and less on that between the two. Despite the fact that numerous observational studies have been carried out to look into the connection between vitamin D deficiency and dyslipidemia, there is still no concrete proof of a causal link between the two diseases. In contrast to large-scale prospective experiments that require significant resources over a long period of time, MR Analyses reveal cause-and-effect relationships in a time-saving and cost-effective manner [33].

In this Mendelian randomized study, we observed evidence of a negative causal relationship between genetically determined serum 25(OH)D levels and serum TG, TC, and LDL-C levels, consistent with the findings of other studies [12]. In contrast, we found no evidence of a causal relationship between serum 25(OH)D levels and HDL-C levels. Jennifer et al. ‘s results showed that serum 25(OH)D levels were not associated with HDL-C levels [12], and Yonghong Li et al. ‘s cohort study showed that individuals whose vitamin D levels decreased year by year tended to have increased levels of TC, LDL-C and TG, and the change of vitamin D level was not correlated with the change of HDL-C level [34], which is consistent with our results.

Although the mechanisms by which vitamin D affects lipid levels remain unknown, several mechanisms have been proposed. To begin, vitamin D can inhibit liver TG synthesis and secretion by stimulating intestinal calcium absorption [35]. It has also been suggested that vitamin D regulates calcium metabolism and increases calcium absorption in the gut, thereby decreasing fatty acid absorption [36]. Second, serum calcium can promote stool fat excretion and bile acid secretion, thus reducing cholesterol levels [37, 38]. Third, increased TG can be caused by high levels of parathyroid hormone (PTH), and 25 (OH) D can inhibit serum PTH secretion [39, 40]. As a result, vitamin D can influence TG concentration by regulating PTH levels. Therefore, a lack of vitamin D may contribute to insulin resistance and impaired B-cell activity, both of which can alter lipoprotein metabolism and cause increased TG levels and decreased HDL-C levels [41]. Finally, vitamin D may influence lipid levels by increasing VLDL receptor gene expression [42].

At the same time, we observed a negative causal relationship between TG, TC and 25(OH)Ds levels. However, Zhe Lu et al. have found a causal relationship between elevated serum TG, LDL, and HDL levels and decreased serum vitamin D levels, which is inconsistent with the findings of this study [15]. Several studies have been conducted to investigate the potential mechanism of vitamin D deficiency caused by dyslipidemia. According to a meta-analysis, the CYP2R1 gene encodes the most important enzyme in vitamin D metabolism, and its mutation is strongly linked to vitamin D deficiency [43]. Another rat study discovered that diet-induced elevations in circulating cholesterol and glucose reduced serum vitamin D levels by inhibiting CYP2R1 expression in the liver. The specific molecular mechanism by which dyslipidemia affects the expression of CYP2R1 is still unknown [44].

The study’s strength is that we used large-scale genetic data to examine the bidirectional causal relationship between vitamin D and lipids using MR Analysis, allowing us to eliminate confounders and reverse causality biases. And we conducted a comprehensive sensitivity analysis to ensure that our results were robust against various assumptions. This study has some limitations as well. First, since the two-sample MR Analysis is predicated on European ancestry, individuals descended from other ancestors may have different relationships; two-sample MR Analysis should be conducted on at least one additional lineage. Second, because IV is extracted from abstraction-level data, nonlinear relationship analysis or subgroup analysis is usually not possible. Furthermore, the MR Analysis can only provide a preliminary determination of the causal relationship between vitamin D and blood lipids, and the specific mechanism by which vitamin D deficiency increases the risk of dyslipidemia requires further investigation.

## 5 Conclusions

In conclusion, our MR Study found evidence of a link between vitamin D deficiency and an increased risk of high TG, high TC, and high LDL-C lipids, as well as a link between TG, TC, and vitamin D. The findings have potential implications for etiology and prevention.

It is critical to prevent dyslipidemia, which can lead to cardiovascular disease, stroke, and diabetes, ultimately leading to increased mortality. Vitamin D deficiency is also linked to an increased risk of developing cancer, autoimmune diseases, cardiovascular disease, type 2 diabetes, and infectious diseases. These findings suggest that serum vitamin D levels can be altered to reduce the risk of dyslipidemia, as well as that dyslipidemia should be treated as soon as possible to avoid vitamin D deficiency.

## Supporting information

S1 Fig Leave-one-out analysis for 25(OH)D on TG. (TIF)

S2 Fig Leave-one-out analysis for 25(OH)D on TC. (TIF)

S3 Fig Leave-one-out analysis for 25(OH)D on HDL-C. (TIF)

S4 Fig Leave-one-out analysis for 25(OH)D on LDL-C. (TIF)

S5 Fig Funnel plots from genetically predicted causal effect between 25(OH)D and TG. (TIF)

S6 Fig Funnel plots from genetically predicted causal effect between 25(OH)D and TC. (TIF)

S7 Fig Funnel plots from genetically predicted causal effect between 25(OH)D and HDL-C. (TIF)

S8 Fig Funnel plots from genetically predicted causal effect between 25(OH)D and LDL-C. (TIF)

S9 Fig Scatter plot of causal effect of Mendel randomized (MR) analysis of serum TG levels on 25(OH)D levels. (TIF)

S10 Fig Scatter plot of causal effect of Mendel randomized (MR) analysis of serum TC levels on 25(OH)D levels. (TIF)

S11 Fig Scatter plot of causal effect of Mendel randomized (MR) analysis of serum HDL-C levels on 25(OH)D levels. (TIF)

S12 Fig Scatter plot of causal effect of Mendel randomized (MR) analysis of serum LDL-C levels on 25(OH)D levels. (TIF)

S13 Fig Leave-one-out analysis for TG on 25(OH)D. (TIF)

S14 Fig Leave-one-out analysis for TC on 25(OH)D. (TIF)

S15 Fig Leave-one-out analysis for HDL-C on 25(OH)D. (TIF)

S16 Fig Leave-one-out analysis for LDL-C on 25(OH)D. (TIF)

S17 Fig Funnel plots from genetically predicted causal effect between TG on 25(OH)D. (TIF)

S18 Fig Funnel plots from genetically predicted causal effect between TC on 25(OH)D. (TIF)

S19 Fig Funnel plots from genetically predicted causal effect between HDL-C on 25(OH)D. (TIF)

S20 Fig Funnel plots from genetically predicted causal effect between LDL-C on 25(OH)D. (TIF)

S1 File SNPs used to analyze the causal relationship between serum 25 (OH) D levels and serum lipids levels. (XLSX)
