# Supplementary figures and images for: The causal relationship between 25-hydroxyvitamin D and serum lipids levels: A bidirectional two-sample mendelian randomization study
Source: PLoS One. 2024 Feb 14;19(2):e0287125. doi: 10.1371/journal.pone.0287125 (PMC10866529; doi:10.1371/journal.pone.0287125)

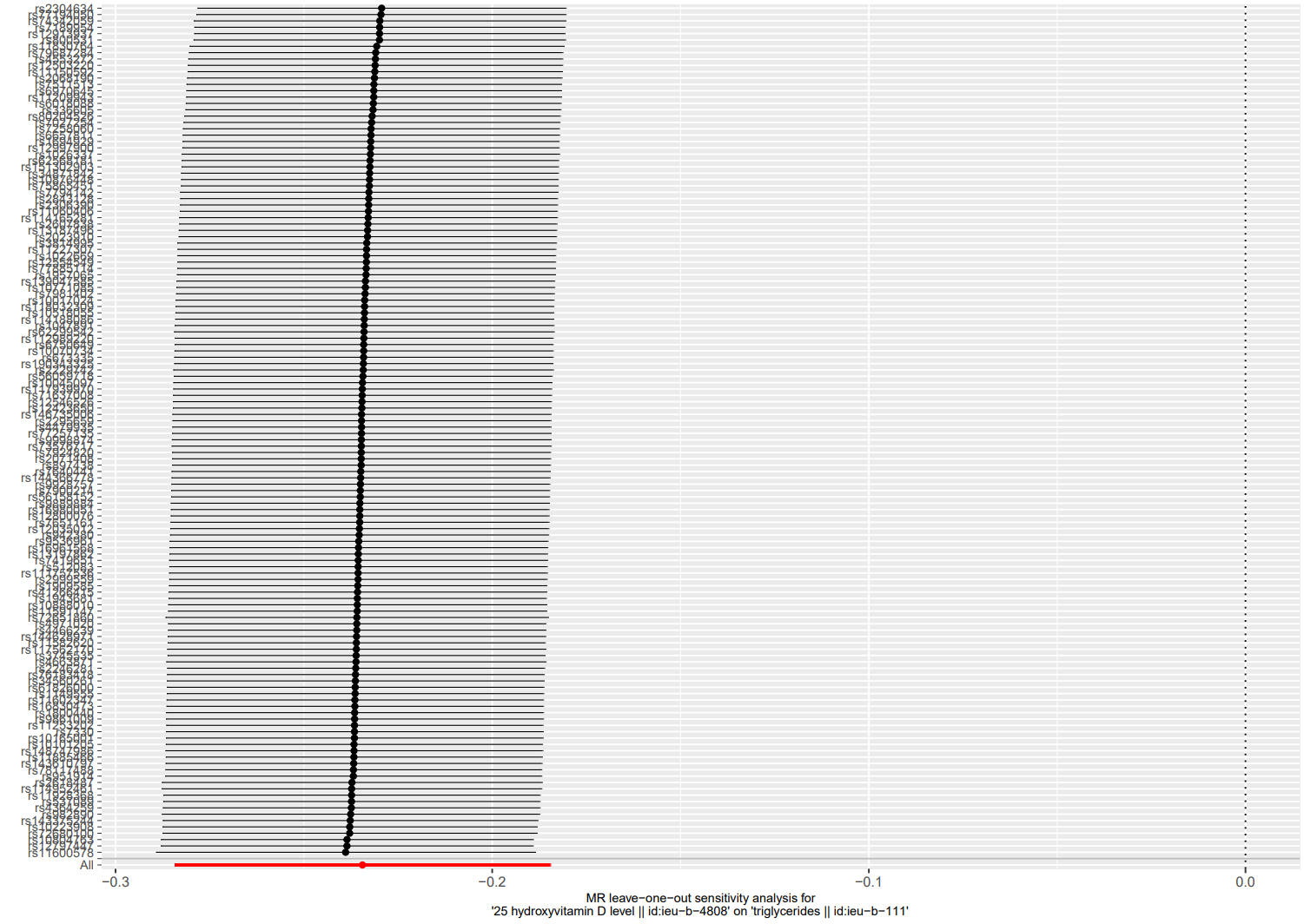

Supplement: S1 Fig — (TIF) [file pone.0287125.s001.tif]

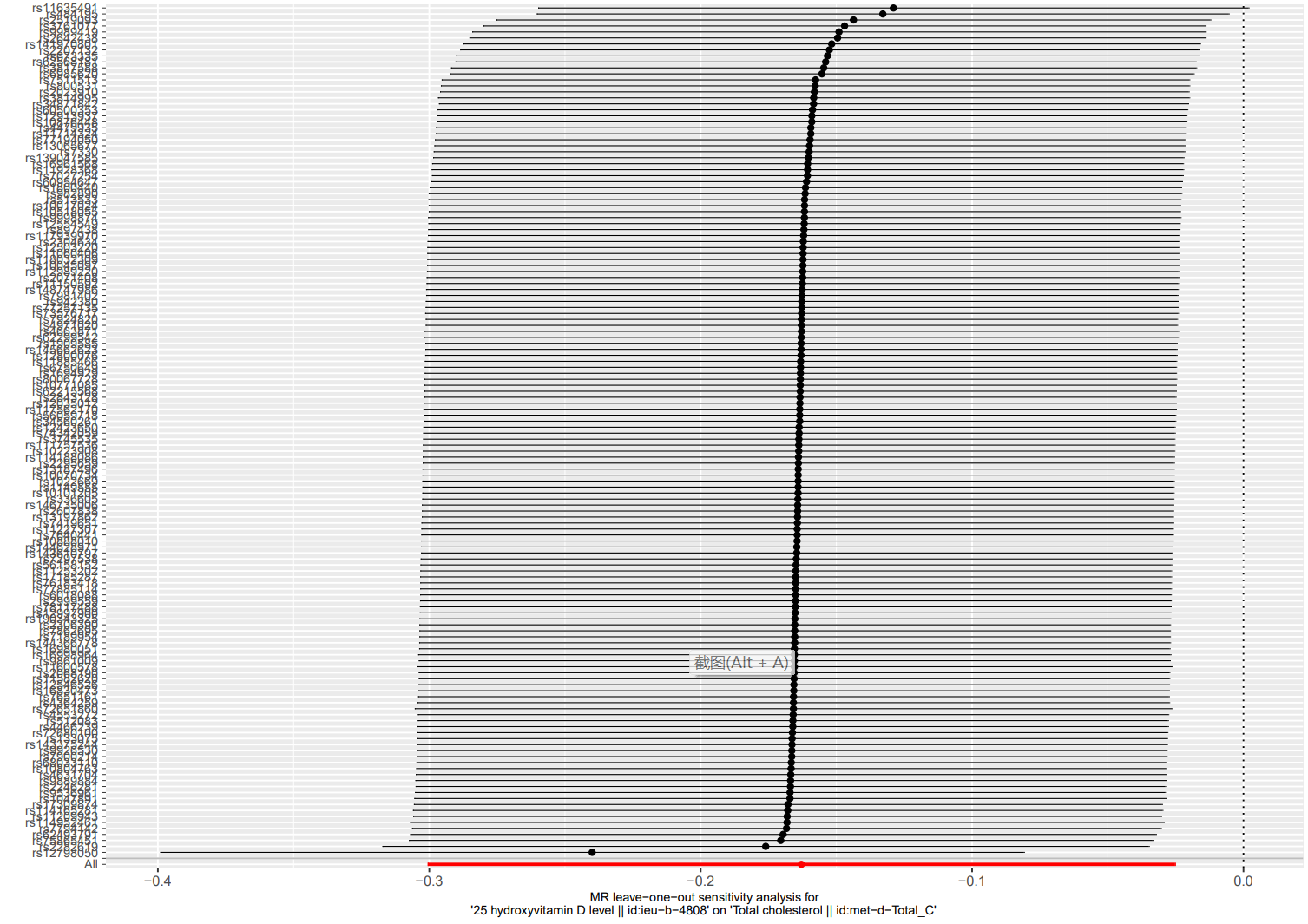

Supplement: S2 Fig — (TIF) [file pone.0287125.s002.tif]

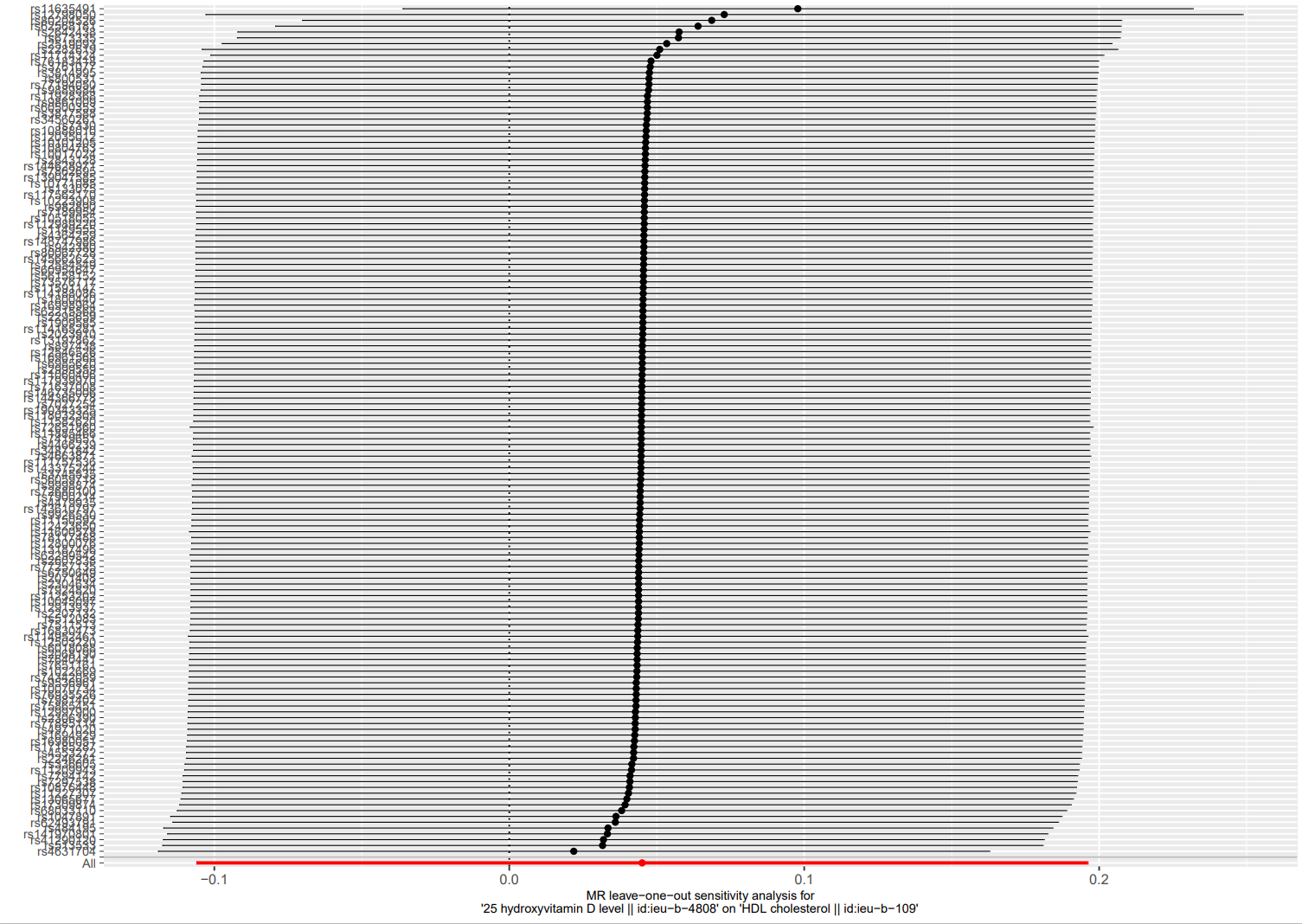

Supplement: S3 Fig — (TIF) [file pone.0287125.s003.tif]

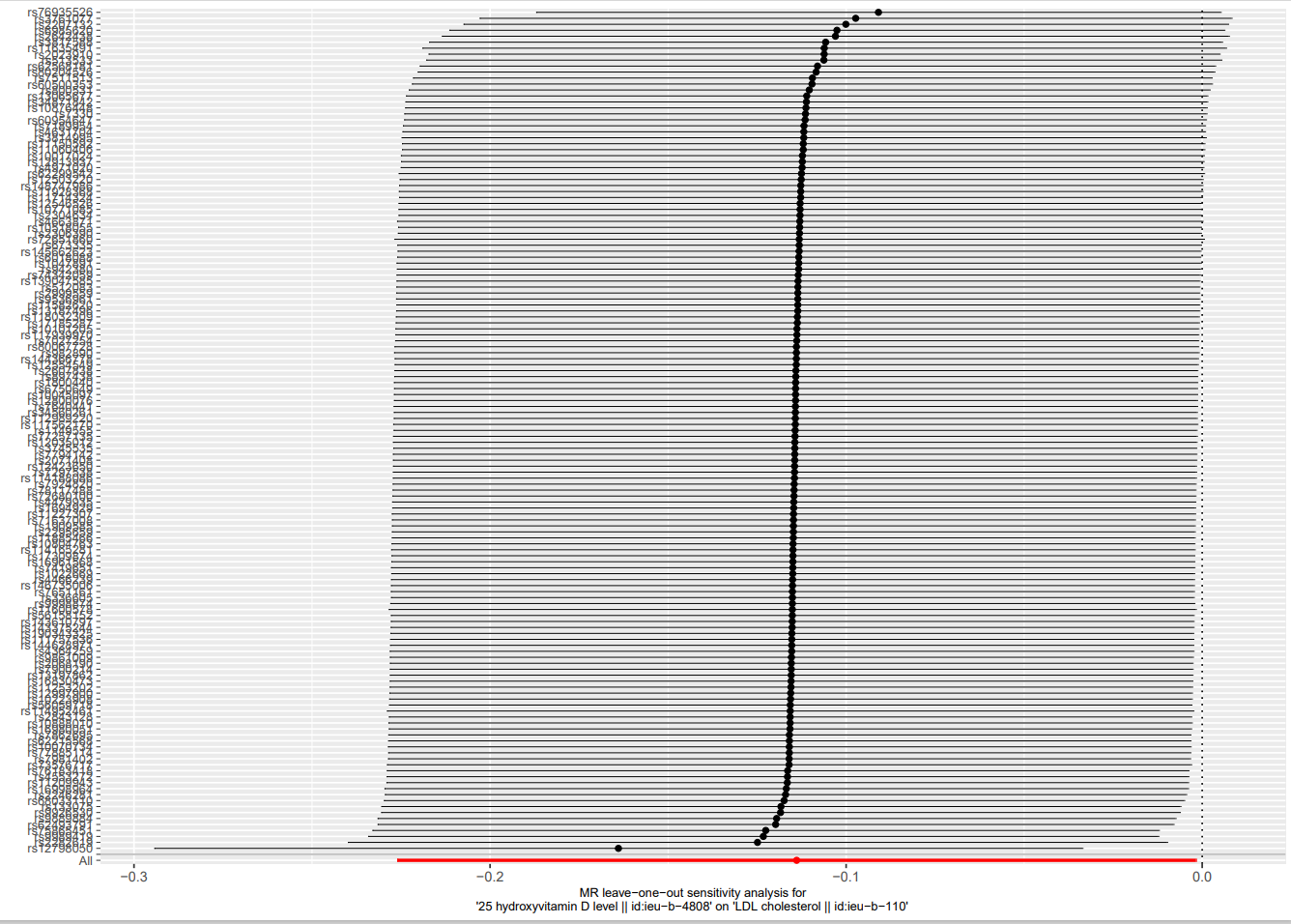

Supplement: S4 Fig — (TIF) [file pone.0287125.s004.tif]

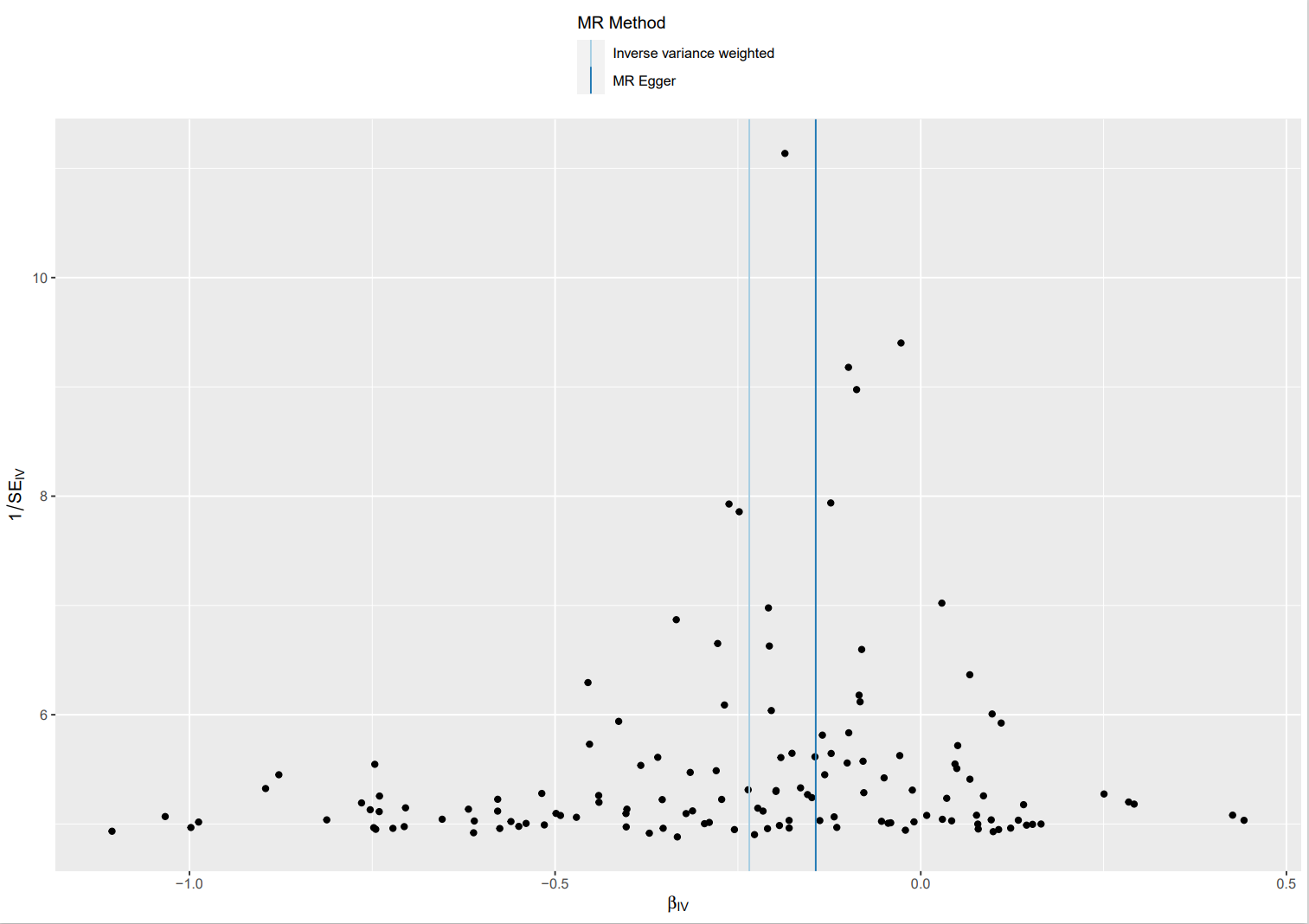

Supplement: S5 Fig — (TIF) [file pone.0287125.s005.tif]

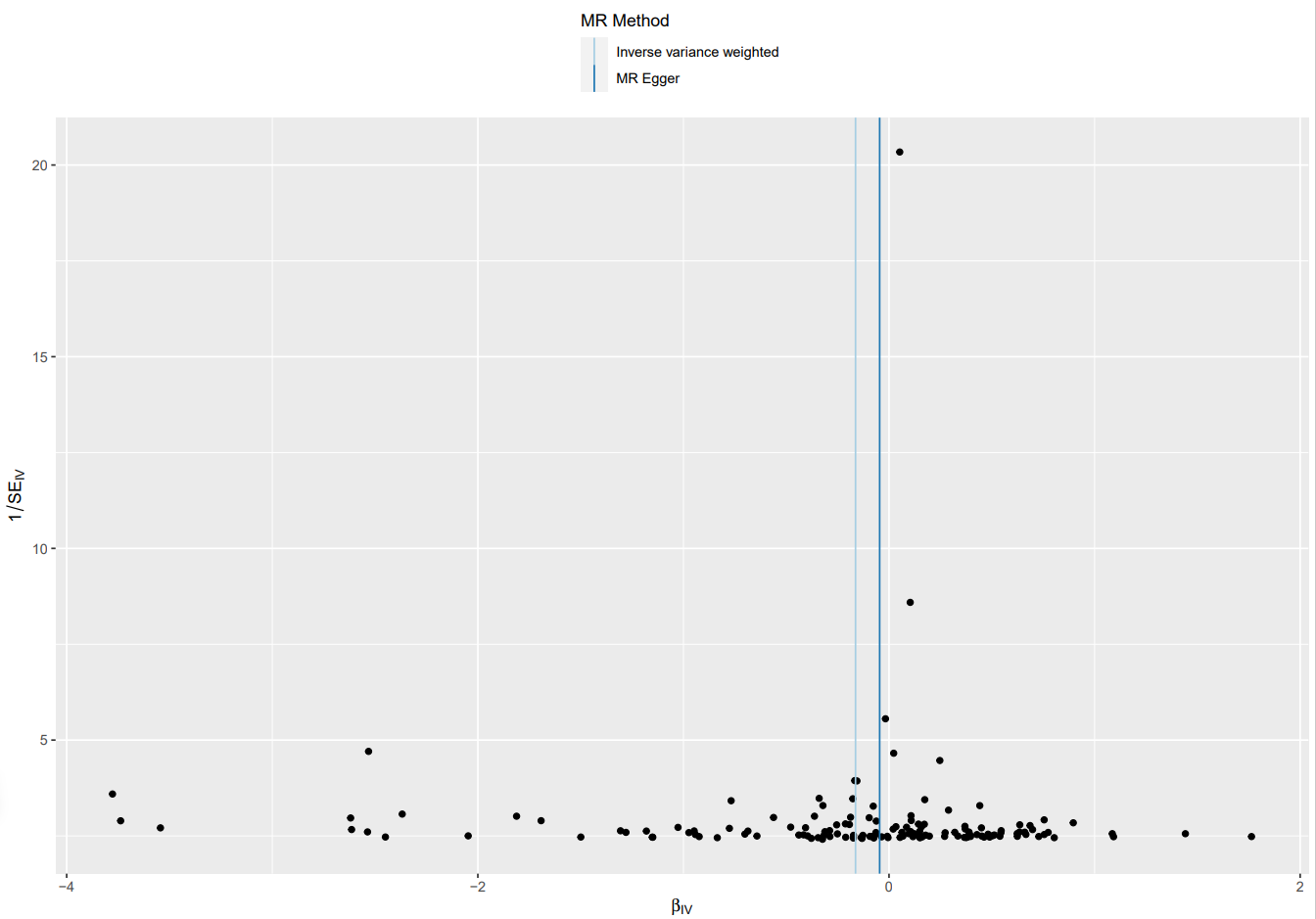

Supplement: S6 Fig — (TIF) [file pone.0287125.s006.tif]

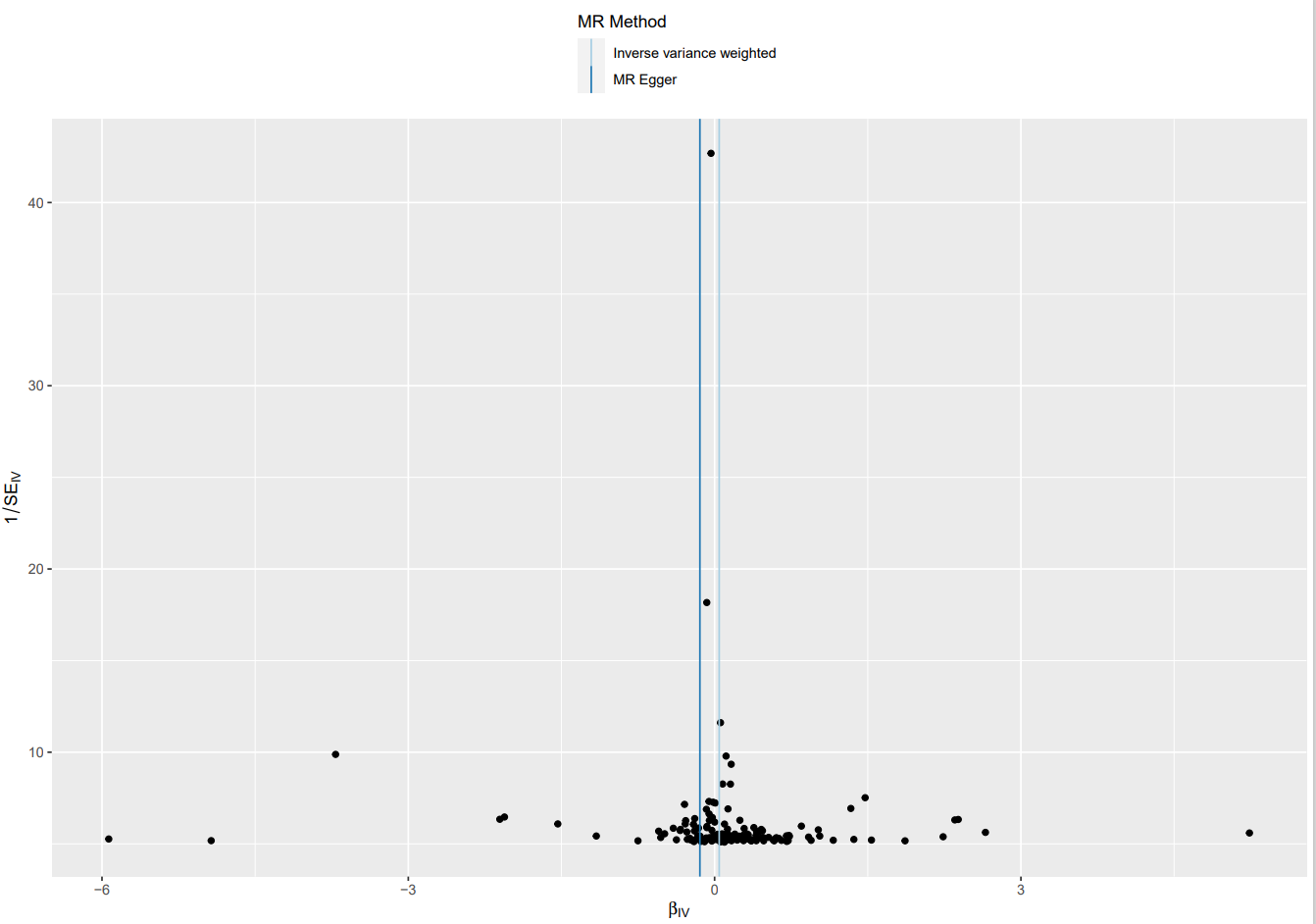

Supplement: S7 Fig — (TIF) [file pone.0287125.s007.tif]

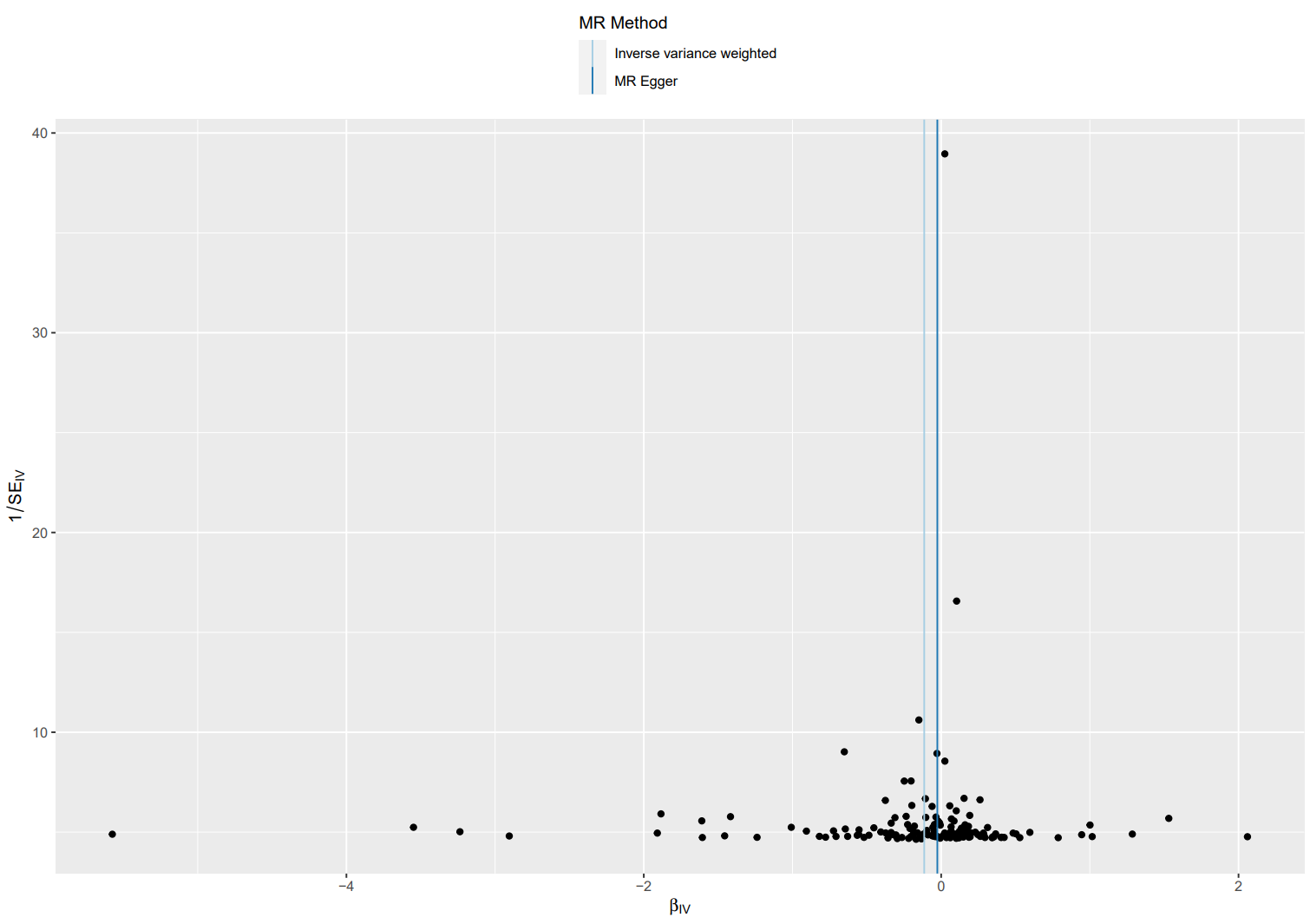

Supplement: S8 Fig — (TIF) [file pone.0287125.s008.tif]

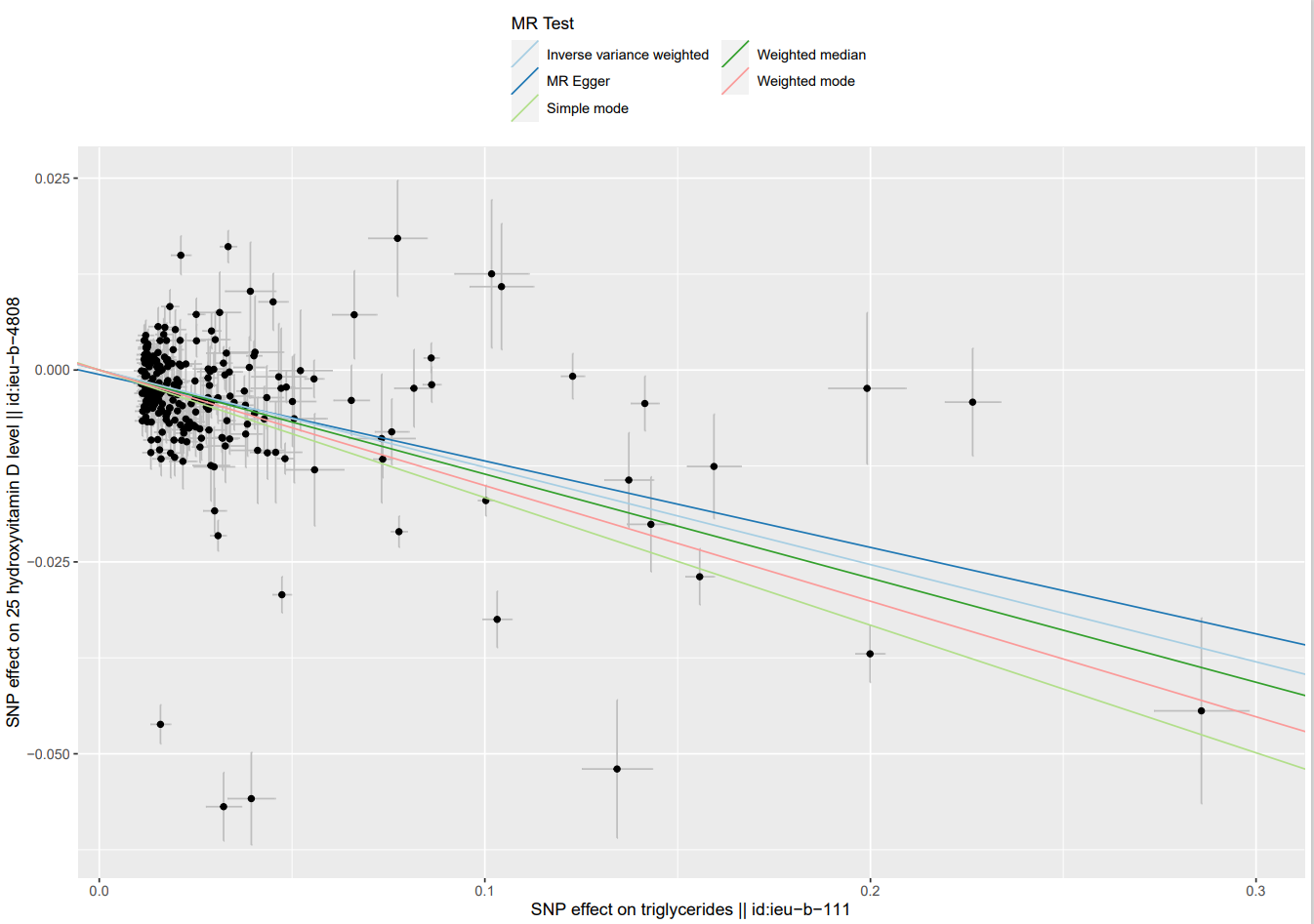

Supplement: S9 Fig — (TIF) [file pone.0287125.s009.tif]

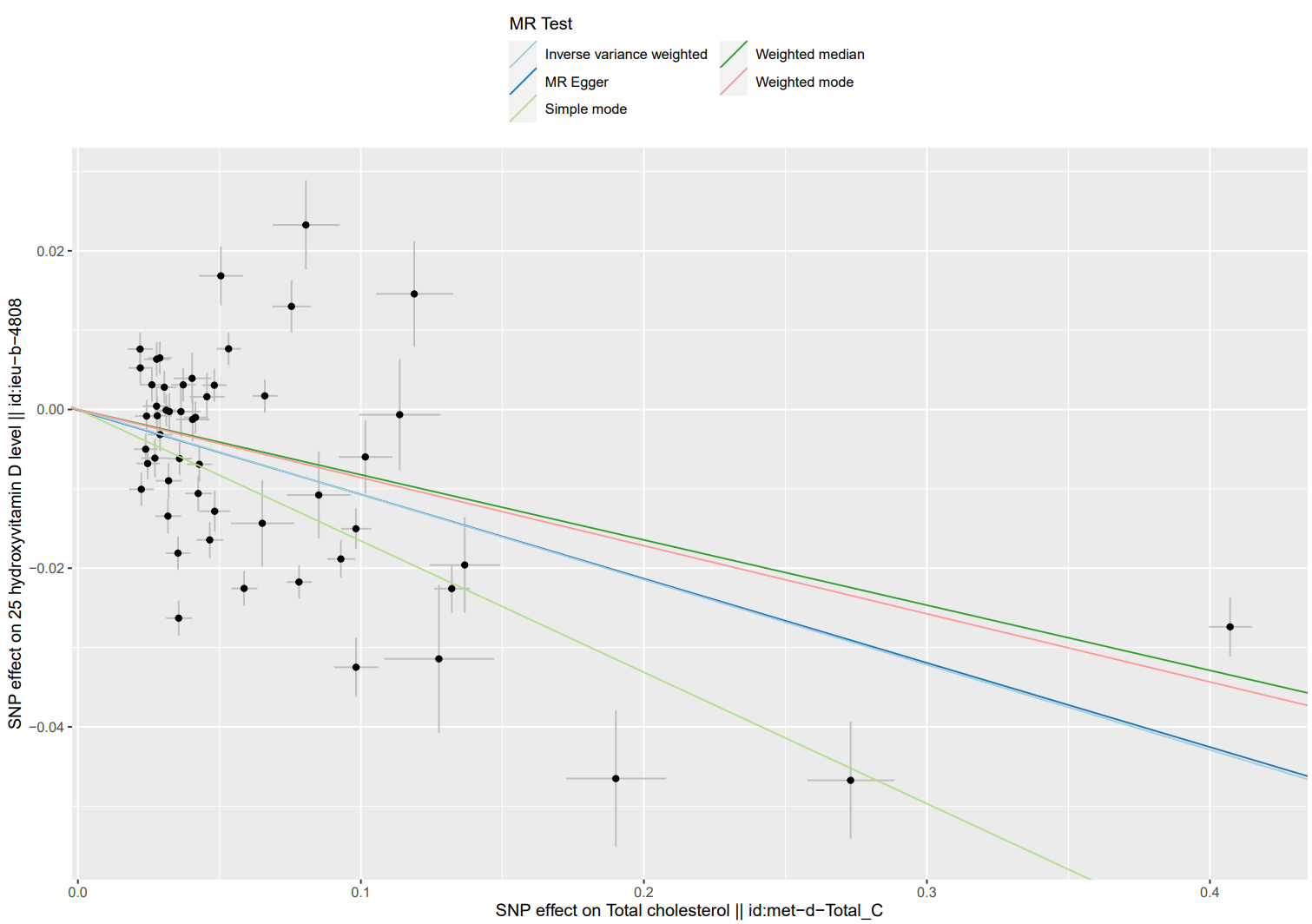

Supplement: S10 Fig — (TIF) [file pone.0287125.s010.tif]

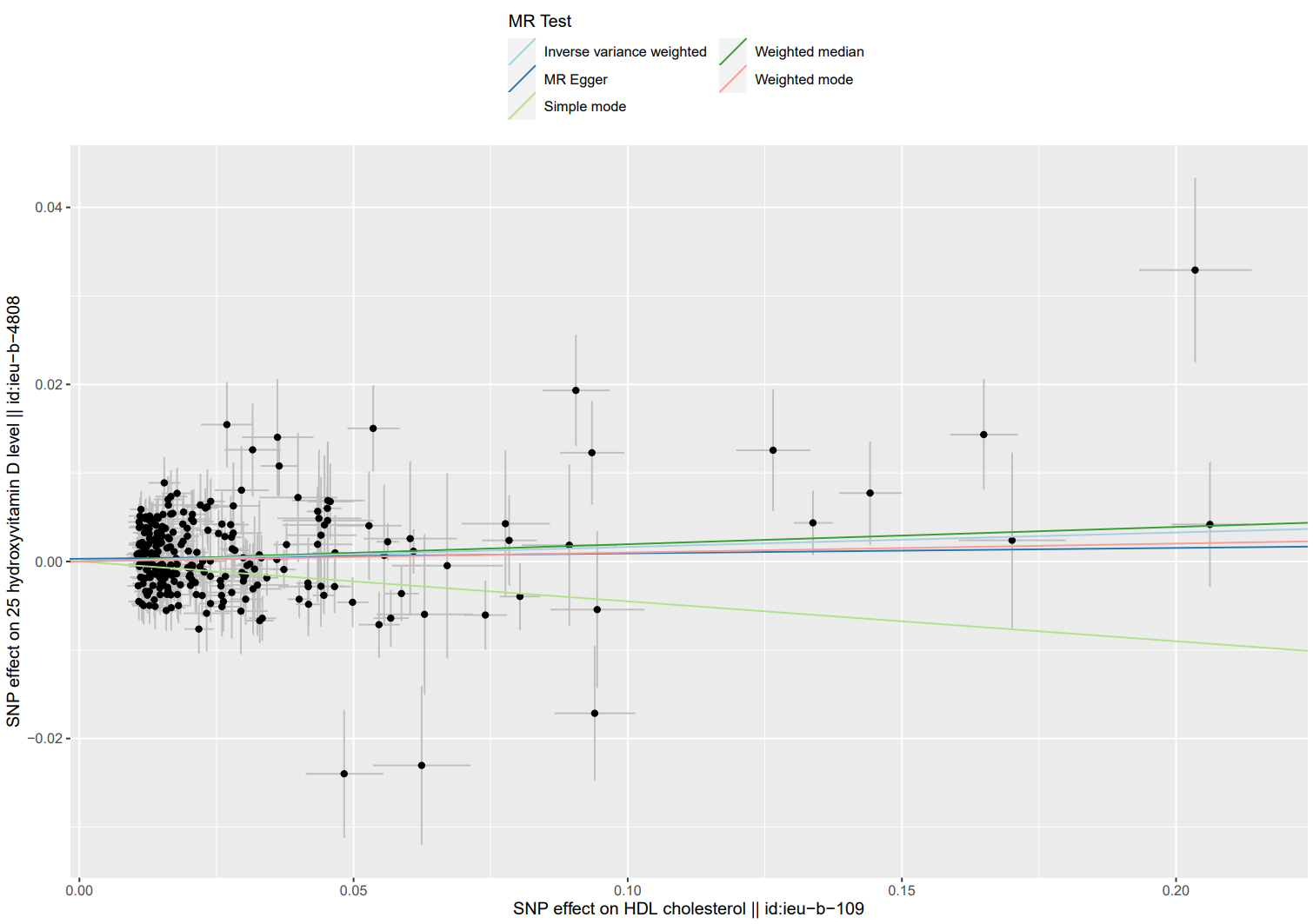

Supplement: S11 Fig — (TIF) [file pone.0287125.s011.tif]

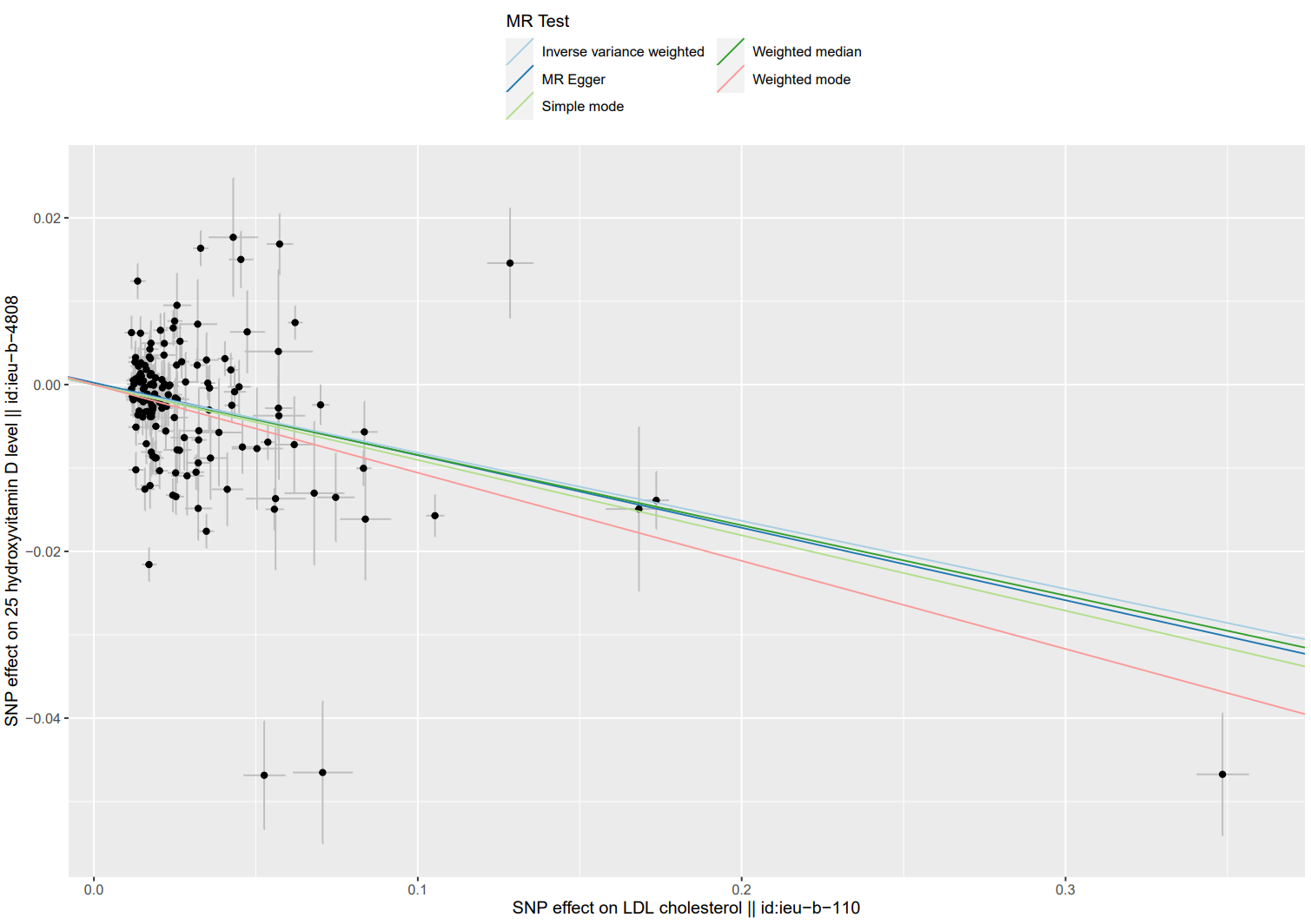

Supplement: S12 Fig — (TIF) [file pone.0287125.s012.tif]

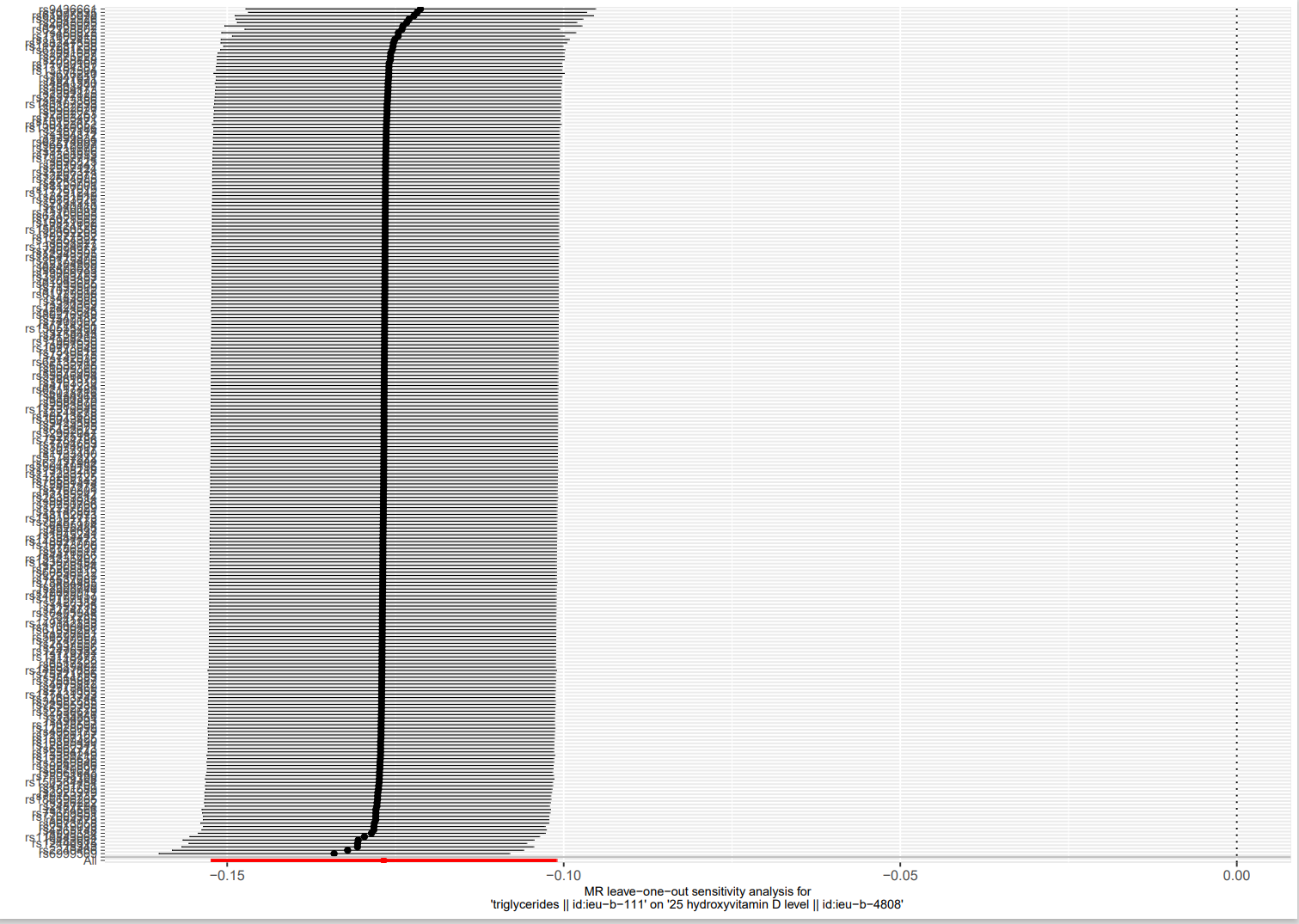

Supplement: S13 Fig — (TIF) [file pone.0287125.s013.tif]

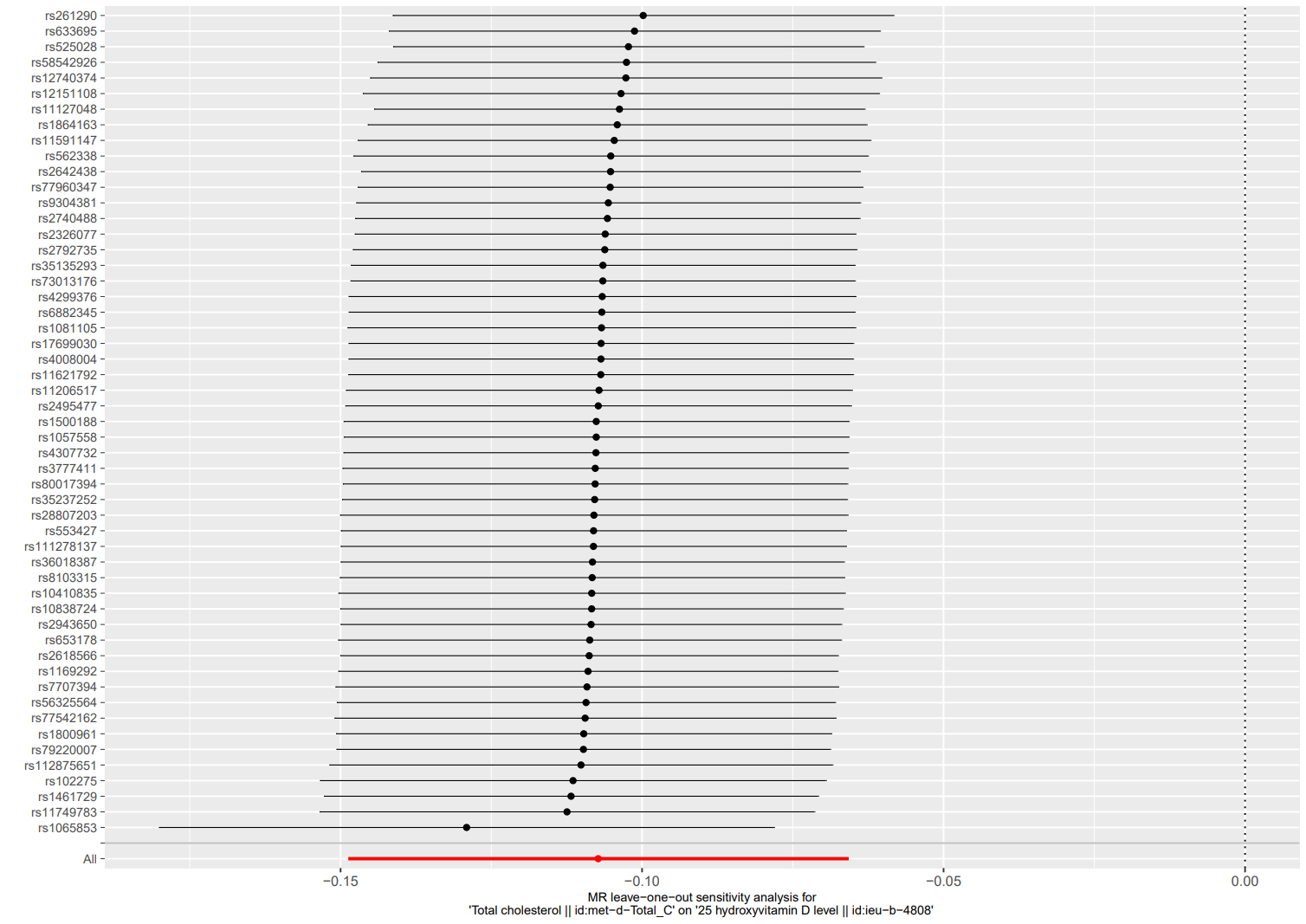

Supplement: S14 Fig — (TIF) [file pone.0287125.s014.tif]

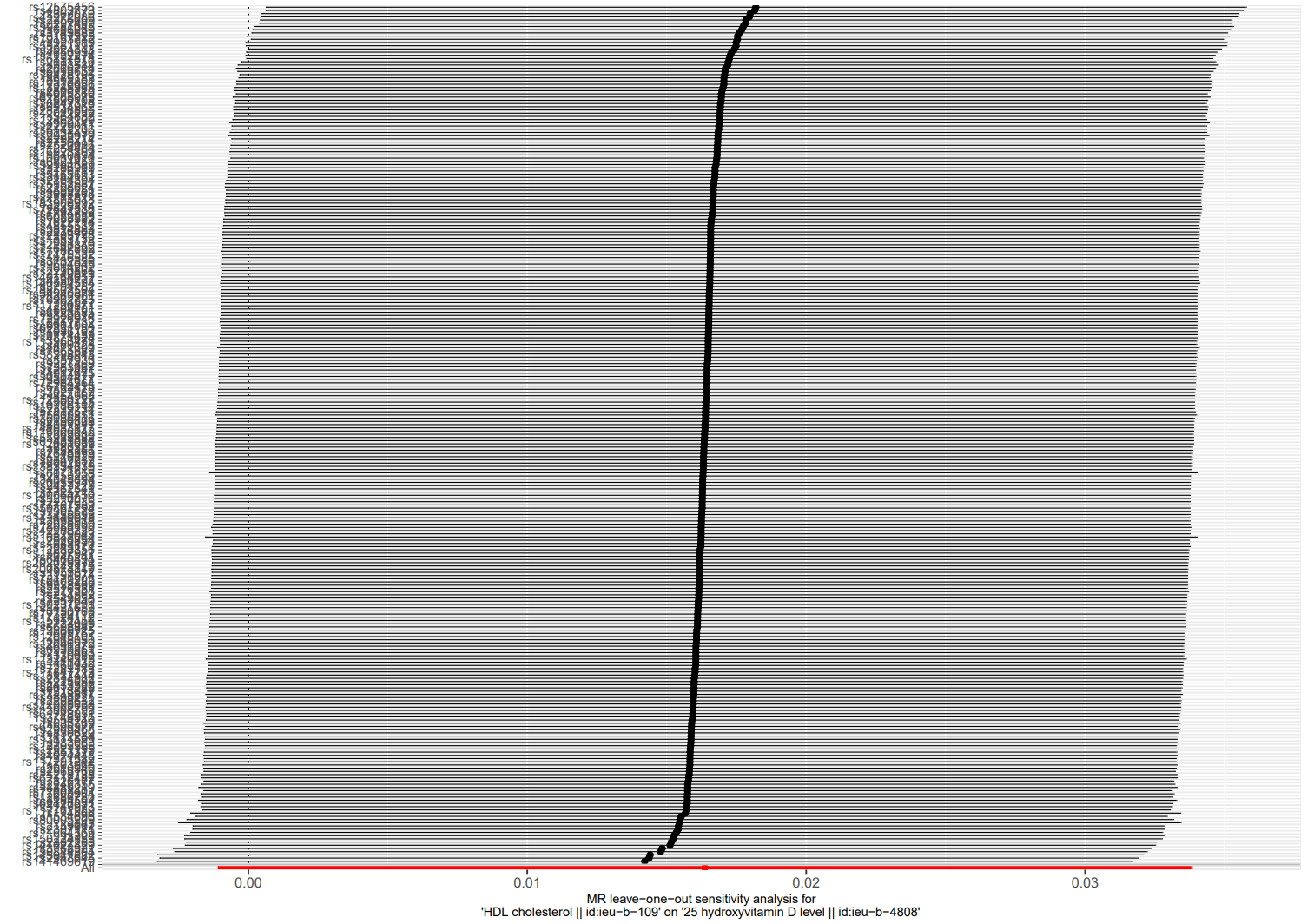

Supplement: S15 Fig — (TIF) [file pone.0287125.s015.tif]

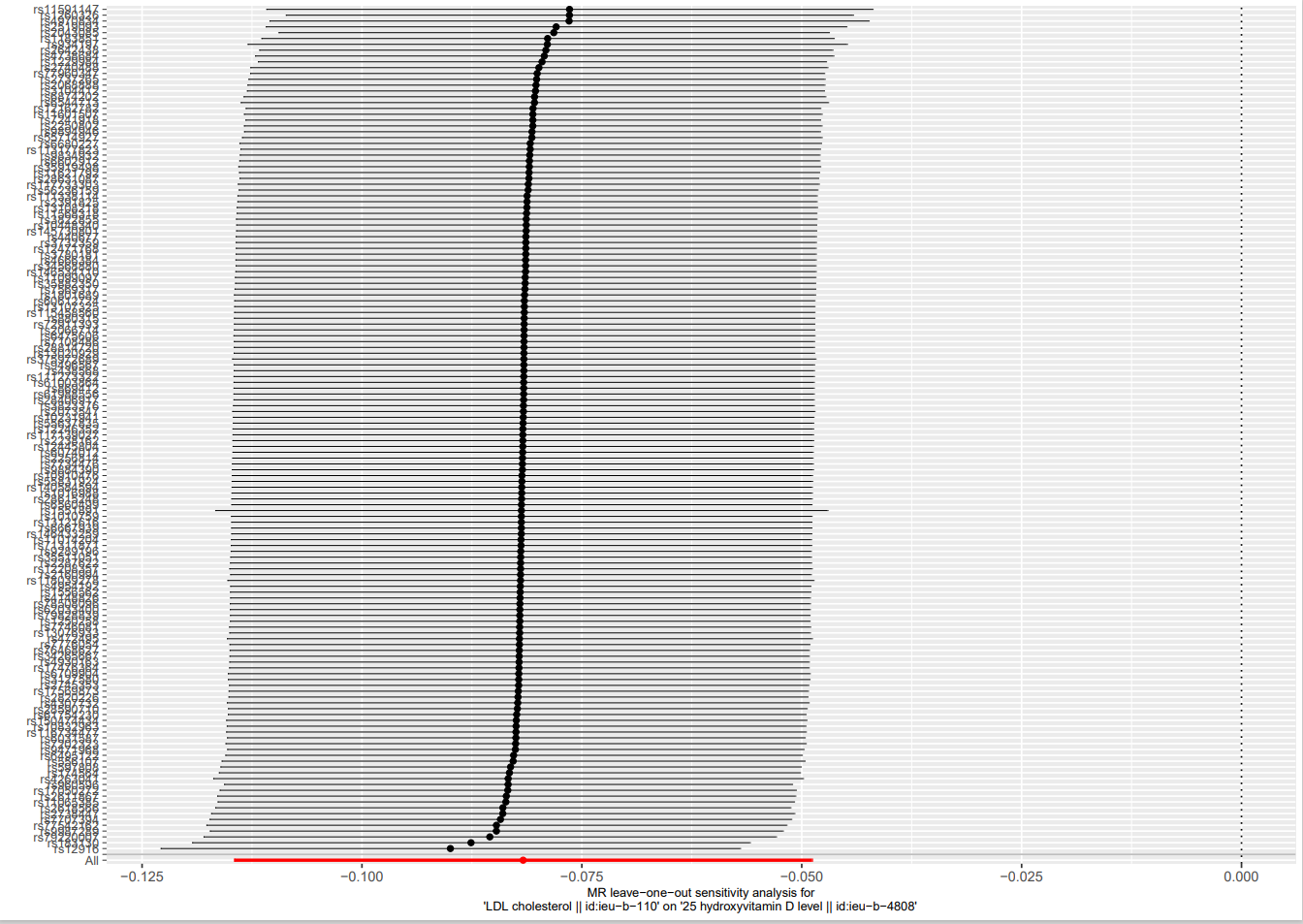

Supplement: S16 Fig — (TIF) [file pone.0287125.s016.tif]

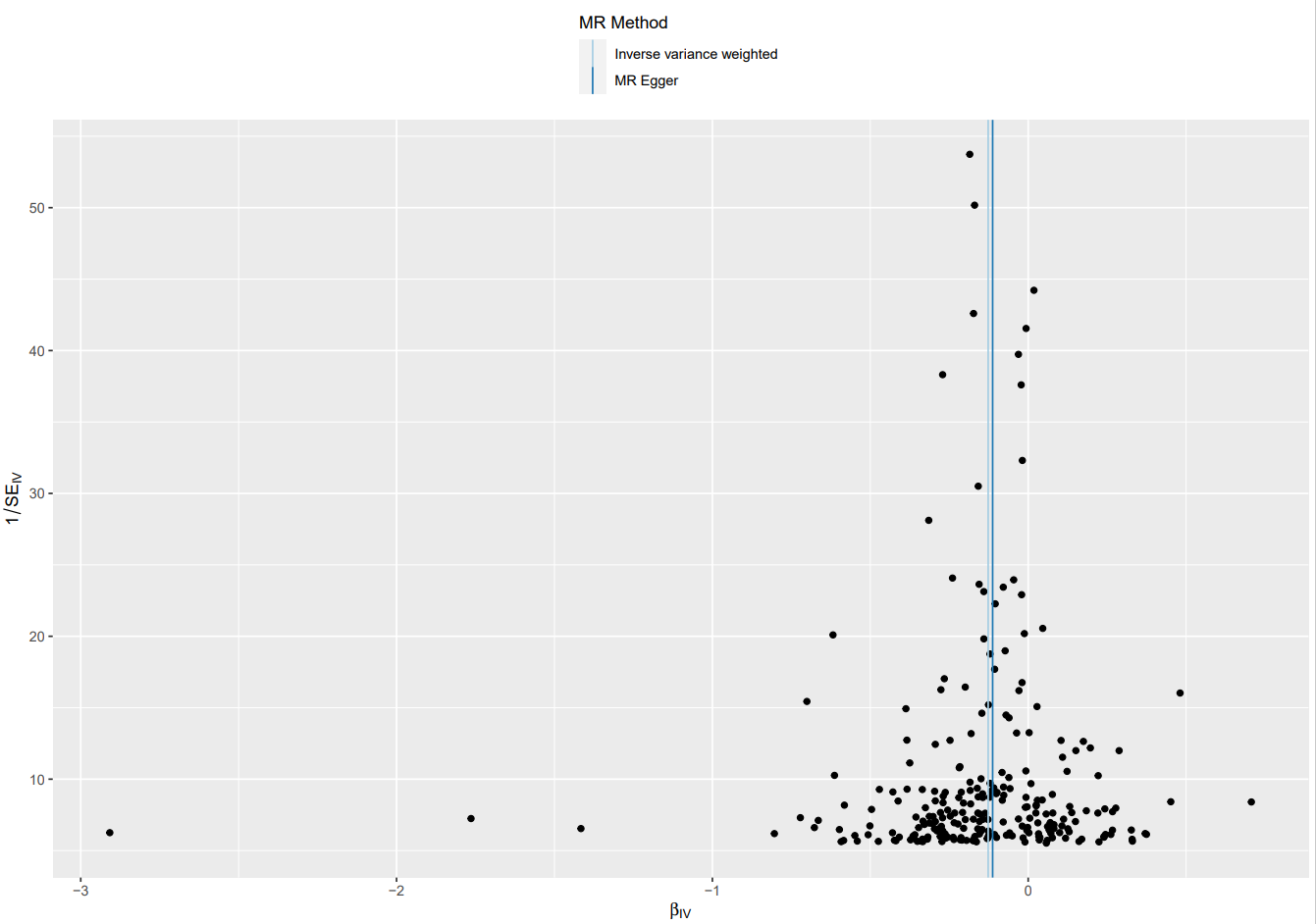

Supplement: S17 Fig — (TIF) [file pone.0287125.s017.tif]

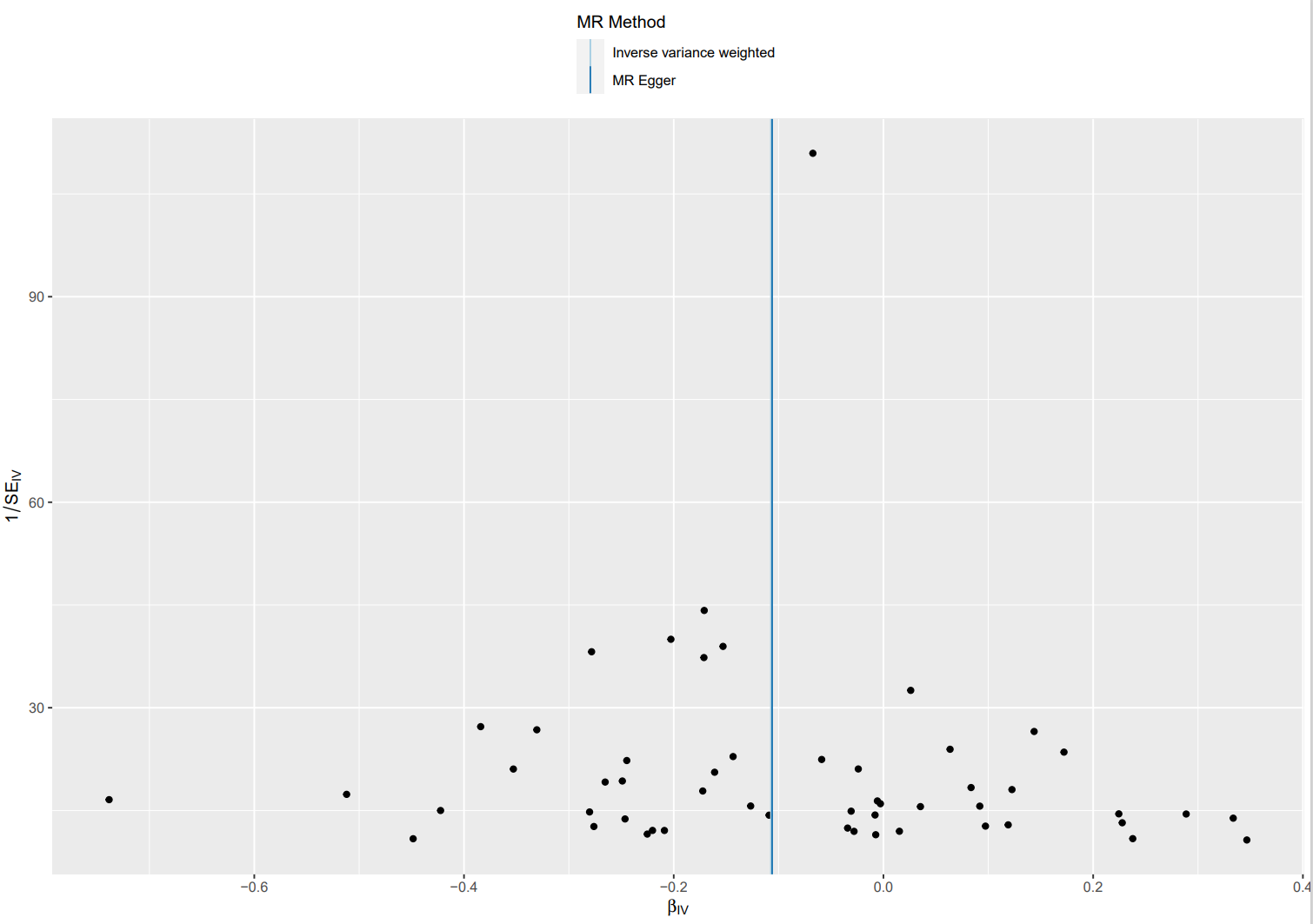

Supplement: S18 Fig — (TIF) [file pone.0287125.s018.tif]

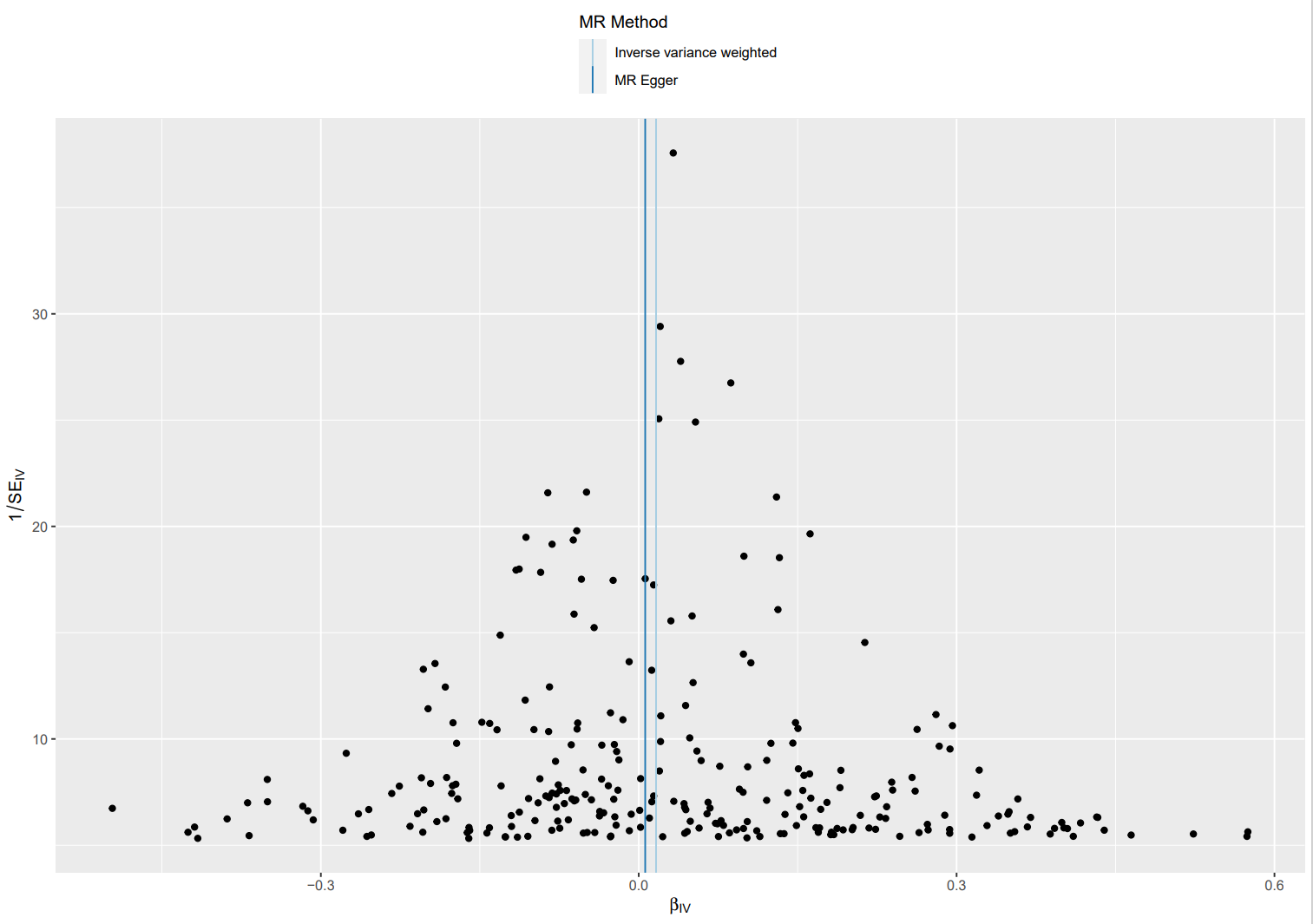

Supplement: S19 Fig — (TIF) [file pone.0287125.s019.tif]

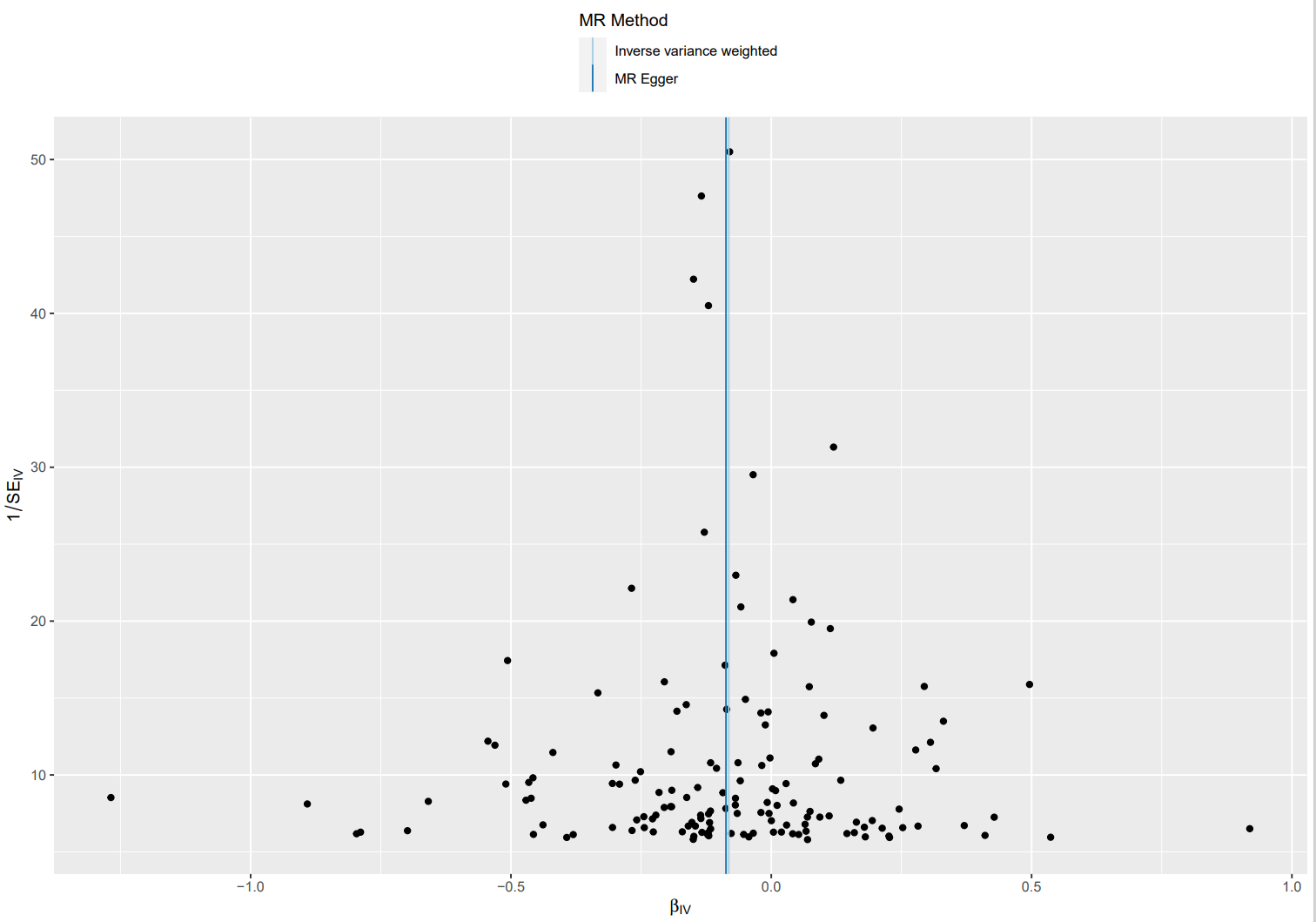

Supplement: S20 Fig — (TIF) [file pone.0287125.s020.tif]
